# Transcriptome of the Australian Mollusc *Dicathais orbita* Provides Insights into the Biosynthesis of Indoles and Choline Esters

**DOI:** 10.3390/md14070135

**Published:** 2016-07-20

**Authors:** Abdul Baten, Ajit Kumar Ngangbam, Daniel L. E. Waters, Kirsten Benkendorff

**Affiliations:** 1 Southern Cross Plant Science, Southern Cross University, Lismore NSW 2480, Australia; abdul.baten@scu.edu.au (A.B.); daniel.waters@scu.edu.au (D.L.E.W.); 2 Marine Ecology Research Centre, School of Environment, Science and Engineering, Southern Cross University, Lismore NSW 2480, Australia; a.ngangbam.10@student.scu.edu.au

**Keywords:** Muricidae, transcriptomics, Tyrian purple, tryptophan, metabolic pathways

## Abstract

*Dicathais orbita* is a mollusc of the Muricidae family and is well known for the production of the expensive dye Tyrian purple and its brominated precursors that have anticancer properties, in addition to choline esters with muscle-relaxing properties. However, the biosynthetic pathways that produce these secondary metabolites in *D. orbita* are not known. Illumina HiSeq 2000 transcriptome sequencing of hypobranchial glands, prostate glands, albumen glands, capsule glands, and mantle and foot tissues of *D. orbita* generated over 201 million high quality reads that were de novo assembled into 219,437 contigs. Annotation with reference to the Nr, Swiss-Prot and Kyoto Encyclopedia of Genes and Genomes (KEGG) databases identified candidate-coding regions in 76,152 of these contigs, with transcripts for many enzymes in various metabolic pathways associated with secondary metabolite biosynthesis represented. This study revealed that *D. orbita* expresses a number of genes associated with indole, sulfur and histidine metabolism pathways that are relevant to Tyrian purple precursor biosynthesis, and many of which were not found in the fully annotated genomes of three other molluscs in the KEGG database. However, there were no matches to known bromoperoxidase enzymes within the *D. orbita* transcripts. These transcriptome data provide a significant molecular resource for gastropod research in general and Tyrian purple producing Muricidae in particular.

## 1. Introduction

**Figure 1:**
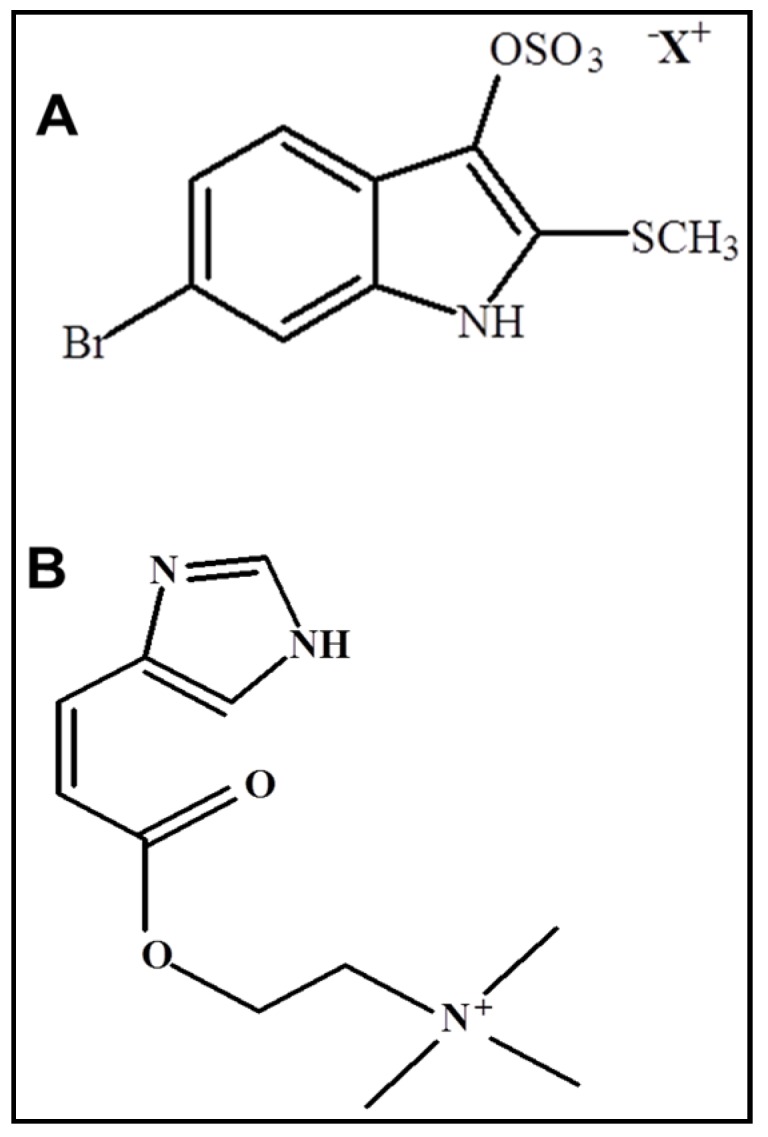
Tyrindoxyl sulfate (**A**), the ultimate Tyrian purple precursor in *Dictahais orbita*, is held as a salt of the choline ester murexine (**B**).

*Dicathais orbita* is well known for the production of the dye Tyrian purple, which is a historically important colourant exclusively produced by the Muricidae family of marine molluscs [1,2]. Tyrian purple is not produced within the mollusc but is formed from oxidative and photolytic reactions from a precursor tyrindoxyl sulfate (Figure 1A), which is stored as a salt of the choline ester murexine (Figure 1B) [3,4]. Once the salt is liberated by an aryl sulfatase enzyme, a range of intermediate brominated indole precursors are produced, which have anticancer and antimicrobial properties [5,6,7,8,9]. The major dye component of Tyrian Purple, 6,6′-dibromoindigo, was the first marine natural product to be structurally elucidated [10]; however, a century later, limited information is available on the biosynthesis or gene regulation of this secondary metabolite.

The “post-genomics era” has seen an expansion in the application of bioinformatics to the fields of transcriptomics, proteomics and metabolomics [11]. Transcriptomics provides information on the genes expressed by an organism under certain circumstances or stages of development, in a particular tissue or cell type [12,13]. Mollusc transcriptome studies have been instrumental in establishing the gene expression events associated with shell formation [14], host parasite interactions [15,16], nervous system function [17,18,19], immune defence [20,21], developmental processes [22,23] and cellular and physiological mechanisms [24,25]. Only a few transcriptome profiling studies have been undertaken on predatory marine neogastropods including the gonadal tissues of *Reishia* (Thais) *clavigera* [23], the mantle, foot, gills and gonadal tissues of *Concholepas concholepas* [26], the alimentary canal and salivary glands of *Colubraia reticulata* [27], the venom glands of *Conus consors* [28] and the venom ducts of *C. tribblei*, *C. lenavati* [29] and *Lophiotoma olangoensis*, a Turrid snail [30].

Transcriptomics can identify genes involved in the biosynthesis of secondary metabolites [31,32]. The Australasian mollusc *D. orbita* is an ideal model species for gene expression studies of biosynthetic pathways that may be involved in the synthesis of biologically active secondary metabolites [3]. A preliminary transcriptome study of *D. orbita* used suppressive subtractive hybridisation to identify genes that were upregulated in the hypobranchial gland, the biosynthetic organ where Tyrian purple is produced [33]. This study confirmed that the hypobranchial gland is a significant site for protein synthesis and regulation, but the only enzyme associated with Tyrian purple production identified was arylsulphatase [33]. However, the study was limited by short read lengths and a low total number of reads. Therefore, the aim of this study was to generate a more comprehensive transcriptome from the hypobranchial glands, prostate glands, capsule glands, albumen glands, and mantle and foot tissues of *D. orbita* (Muricidae Neogastropoda) and then search these transcriptomes for potential metabolic pathways that could contribute to indole and choline ester biosynthesis using the Kyoto Encyclopedia of Genes and Genomes (KEGG) PATHWAYS database.

## 2. Results and Discussion

### 2.1. De Novo Transcriptome Assembly

Transcripome sequencing across the six different tissue types (hypobranchial glands, prostate glands, capsule glands, albumen glands, mantle and foot tissues) resulted in approximately 221 million sequencing reads (Table 1). Raw sequencing reads in FASTQ format were first checked for quality using FASTQC [34] followed by removal of adapter sequences, poly-N stretches and low quality (Phred score < 20) reads using the BBDuck module of the BBMap software package (version 34_90, http://sourceforge.net/projects/bbmap), which resulted in 201 million high quality reads. Table 1 shows the number of raw and quality controlled sequencing reads for all the tissues.

High quality reads were de novo assembled into 219,437 contigs using CLC Genomics server (version 4.9, CLC Bio, Aarhus, Denmark) (Table 2). Transdecoder (version 2.0.3, http://transdecoder.github.io/) identified 76,152 contigs that contained candidate-coding regions that were used for annotation and further downstream analysis.

### 2.2. Transcriptome Annotation

**Figure 2:**
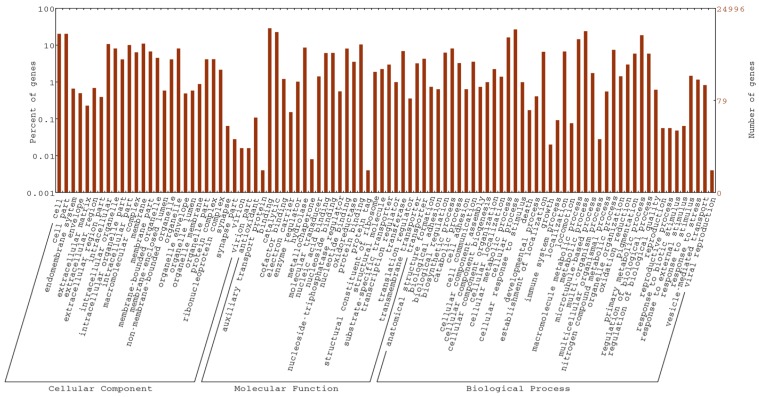
The proportion and number of *Dicathais orbita* contigs assigned to gene ontology (GO) terms from biological process, cellular component and molecular function. Biological process was the most highly represented GO category followed by cellular component and molecular function.

Basic Local Alignment Search Tool (BLAST) analysis was performed using 76,152 contigs with Open Reading Frames (ORF)s against non-redundant protein database National Center for Biotechnology Information (NCBI) Nr (Mollusc specific proteins), Swiss-Prot and KEGG protein databases. Overall 28,364 contigs (~37%) had significant BLAST hits (e value 11 × 10^−5^). The *D. orbita* contig BLAST hit rate was in a similar range to other whole mollusc genome/transcriptome studies with hits ranging from 25% to 40% [35]. A total of 24,996 contigs were assigned to various cellular components, molecular function and biological process gene ontology (GO) categories, as shown in Figure 2. General cell and cell parts were the most frequent subcategories of the cellular components ontology category, while binding and cellular process was the most represented subcategory of molecular function and biological process. Analysis of KEGG pathways showed the largest number of contigs were involved in metabolic pathways (914 contigs), followed by biosynthesis of secondary metabolites (304 contigs) and microbial metabolism in diverse environment (173 contigs) (Supplementary Table S1). Previous studies have revealed a diversity of bacterial symbionts in *D. orbita* tissues [36,37]. However, we checked the overrepresented *k*-mers generated in the quality control phase of RNAseq reads and confirmed that these are mostly mitochondria RNA rather than bacterial (<0.1%).

### 2.3. Tryptophan Metabolism and Phenylalanine, Tyrosine, Tryptophan Biosynthetic Pathways

**Figure 3:**
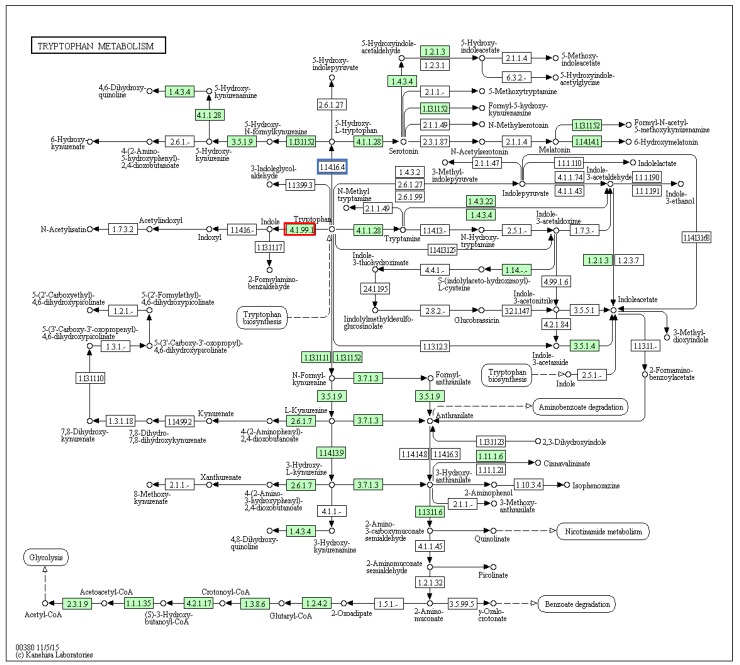
Tryptophan metabolism pathway with matches to *Dicathais orbita* contigs filled in green. The match to a tryptophanase relevant to indole biosynthesis is highlighted by the red box, whereas the tryptophan 5-monoxygenase that was not detected in our transcriptome is highlighted in a blue box.

Specific searches in the KEGG PATHWAY database [38] were undertaken to identify genes potentially involved in the biosynthesis of Tyrian purple precursors and choline esters. Indoles like tyrindoxyl sulfate are thought to be derived from tryptophan metabolism, and we identified 28 enzyme contigs mapped to 35 reactions in the tryptophan metabolism pathway (Figure 3; note that there are multiple KEGG enzyme (EC) numbers for some enzymes, and they can occur at different positions in the pathways, generating more matches to reactions than the number of matching contigs; this applies to all other pathways below). The list of 28 mapped contigs with the KEGG orthology assignment is provided in Supplementary Table S2.

The annotated genomes of only three other molluscs are available for comparison in the KEGG PATHWAYS database, the gastropod *Lottia gigantea*, cephalopod *Octopus bimaculoides* and the bivalve *Crassostrea gigas*. Nearly all the genes involved in tryptophan metabolism found in *D. orbita* (Figure 3) were identical to those found in the other three molluscs (Figure S1). However, an important point of difference is that unlike these other molluscs, the *D. orbita* transcriptome contained tryptophanase (4.1.99.1; Figure 3), which converts tryptophan to indole. Consistent with the other molluscs such as *L. gigantea* and *C. gigas*, *D. orbita* expresses aromatic-l-amino-acid decarboxylase (4.1.1.28; Figure 3) that converts tryptophan to tryptamine. However, we did not detect a transcript for tryptophan 5-monooxygenase (1.14.16.4), which converts tryptophan to 5-hydroxy-l-tryptophan, despite the presence of aromatic-l-amino-acid decarboxylase (4.1.1.28) involved in the production of serotonin. As the other three molluscs all contain matches to tryptophan 5-monoxygenase in their genomes (Figure S1), it is possible that this gene is also present in *D. orbita* but was not detected in our transcriptome due to low expression. Nevertheless, it appears likely that *D. orbita* diverts the conversion of tryptophan away from 5-hydroxy-l-tryptophan in favour of indoles, to facilitate Tyrian purple precursor production by higher expression of the tryptophanase gene.

In the *D. orbita* transcriptome, we found no match in the reaction pathway for the conversion of indole to indoxyl, a precursor to indoxyl sulphate (Figure 3, 1.14.16). However, in bacteria cytochrome P450 enzymes [39,40], and/or mono- or dioxygenases [41,42], are involved in the formation of indoxyl sulfate and indigo. We found matches to two cytochrome P450 enzymes, as well as a monoxidase and several dioxygenases in the *D. orbita* transcriptome (Supplementary Table S2). Our recent studies have also revealed numerous *Vibrio* sp. that have the ability to synthesize indoles in the Tyrian purple producing tissues of *D. orbita* [36,37] and these may provide a supplementary source of indoles for Tyrian purple production.

**Figure 4:**
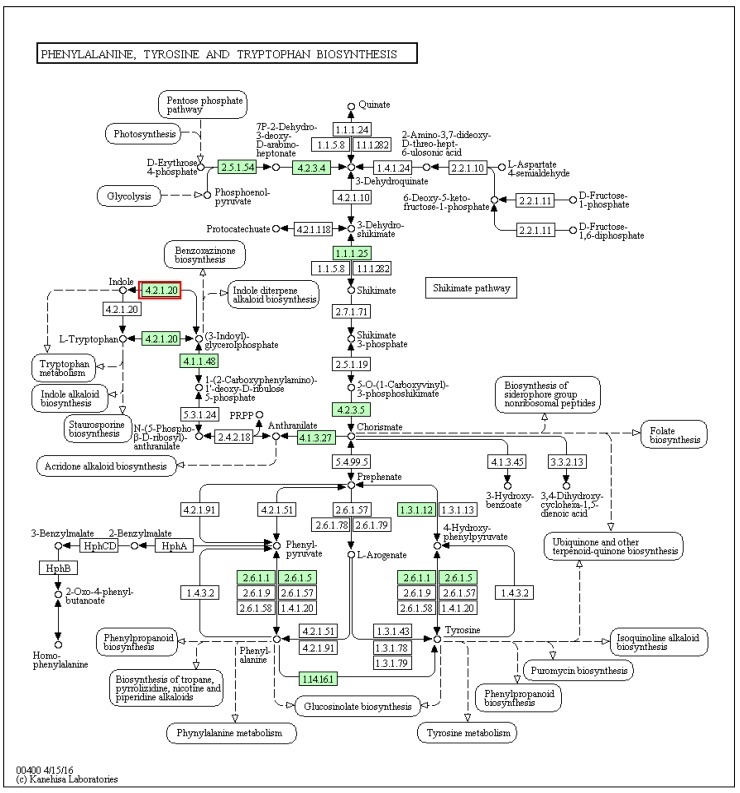
Phenylalanine, tyrosine and tryptophan biosynthetic pathways showing matches to *Dicathais orbita* contigs highlighted in green, with tryptophan synthase highlighted in the red box.

In the phenylalanine, tyrosine and tryptophan biosynthetic pathway (Figure 4), there was a match in the *D. orbita* transcriptome to tryptophan synthase alpha chain (4.2.1.20; Figure 4), which converts tryptophan to indoles and vice versa, the initial precursors for Tyrian purple biosynthesis [3,43,44]. This tryptophan synthase was only found in the *D. orbita* transcriptome and not found in the annotated genomes of *L. gigantea*, *O. bimaculoides* and *C. gigas*. Most of the genes involved in phenylalanine, tyrosine and tryptophan biosynthesis in *D. orbita* were found to be different to the other three molluscs and this Neogastropoda had more matches to these aromatic amino acid biosynthesis reactions (14 genes) compared to *L. gigantea* (five genes), *O. bimaculoides* (five genes) and *C. gigas* (five genes) (Figure S2).

### 2.4. Sulfur, Cysteine and Methionine Metabolisms Pathway in Dicathais orbita

**Figure 5:**
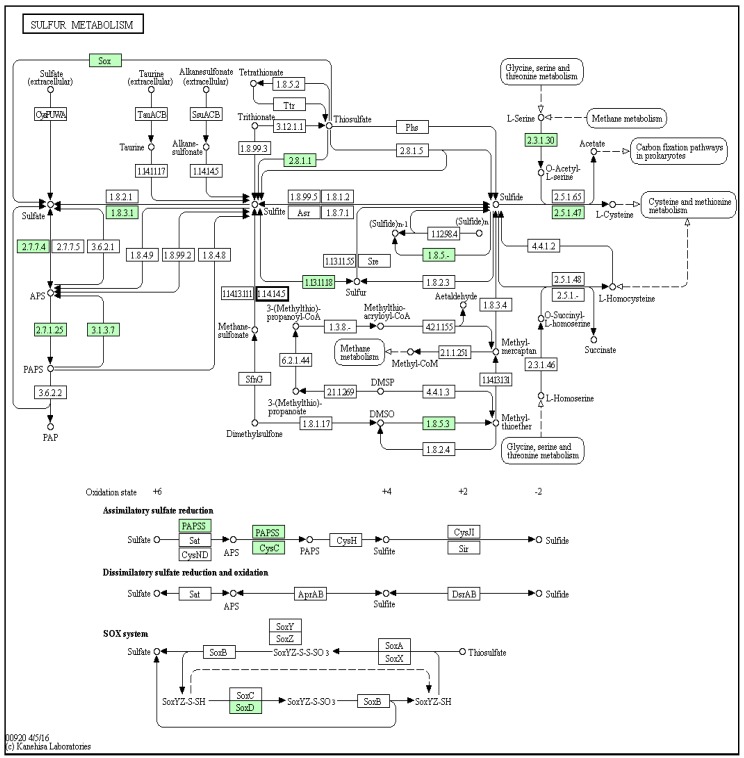
Sulfur metabolism pathway with matches to *Dicathais orbita* contigs highlighted in green; there was no match to dimethyl-sulfide monooxygenase in our transcriptome (**blue** box).

Tyrindoxyl sulphate contains a methane thiol group at the 2′ position of the indole ring (Figure 1A). Examination of the *D. orbita* transcripts with reference to the sulfur metabolism pathway revealed that most of the genes involved in *D. orbita* sulfur metabolism were found to be similar to the other three molluscs. Enzymes like dimethyl-sulfide monooxygenase (1.14.13.131; Figure 5) that produces methyl mercaptan from dimethyl disulphide were not detected in any of the molluscs, including *D. orbita*. Overall, the number of genes detected in the *D. orbita* sulfur metabolism pathway (15 genes, Figure 5) was only slightly higher than the number identified in the genome of *L. gigantea* (12 genes), *O. bimaculoides* (12 genes) and *C. gigas* (14 genes) (Figure S3).

**Figure 6:**
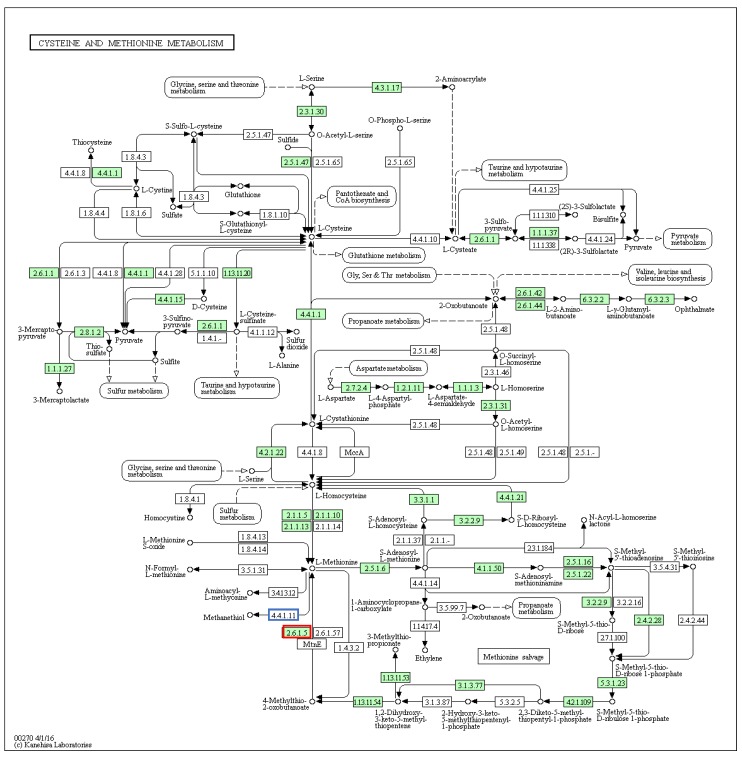
Cysteine and methionine metabolism pathways showing matches to *Dicathais orbita* contigs highlighted in green, including tyrosine aminotransferase (**red** box), but no match was found to methionine-gamma-lyase (**blue** box).

The metabolism of sulfur containing amino acids provides another possible source of the methanethiol group in tyrindoxyl sulfate. Enzyme matches in the cysteine and methionine metabolism pathways (Figure 6) indicate that *D. orbita* has the ability to produce 3-methylthioproprionate, 3-mercaptopyruvate and thiosulfate, but no match was found to methionine-gamma-lyase (4.4.1.11; Figure 6), which converts the amino acid l-Methionine directly into methanethiol. Nevertheless, we did detect a transcript for tyrosine aminotransferase (2.6.1.5; Figure 6), which may play a role in placing the methane thiol onto the aromatic indole ring. Tyrosine aminotransferase genes were also found in the *Lottia*, octopus and oyster genomes. Overall, the *D. orbita* transcriptome had more matches to enzymes in the cysteine and methionine metabolism pathway (41 genes) compared to *L. gigantea* (32 genes), *O. bimaculoides* (30 genes) and *C. gigas* (33 genes) (Figure S4). This ability to metabolise sulfur from various sources is consistent with the biosynthesis of indole mercaptans in Muricidae.

### 2.5. Bromoperoxidase Enzymes

Tyridoxyl sulfate is a 6-brominated indole derivative (Figure 1A), and bromoperoxidase activity has been detected in the hypobranchial glands of *D. orbita* [45] and other Muricidae species [46]. Consequently, a search was undertaken for bromoperoxidase genes by aligning *D. orbita* transcripts against known bromoperoxidase genes using BLAST (e value 1 × 10^−3^). However, no evidence of matches to bromoperoxidase genes was found using these sequences. This is consistent with a previous study that examined the transcripts that were up-regulated in the *D. orbita* hypobranchial glands relative to other *D. orbita* tissues, which also found no matches to bromoperoxidase genes [33]. There appears to be no previous reports of bromoperoxidase coding genes or transcripts in any gastropods or other molluscs. However, recent metagenomic analyses of *D. orbita* hypobranchial glands revealed the presence of bacterial symbionts known to produce bromoperoxidase enzymes [36]. Our *D. orbita* transcriptome data therefore supports the suggestion that symbiotic bacteria play a role in the biosynthesis of Tyrian purple.

### 2.6. Dicathais Orbita Glycerophospholipid and Histidine Metabolism Pathway

**Figure 7:**
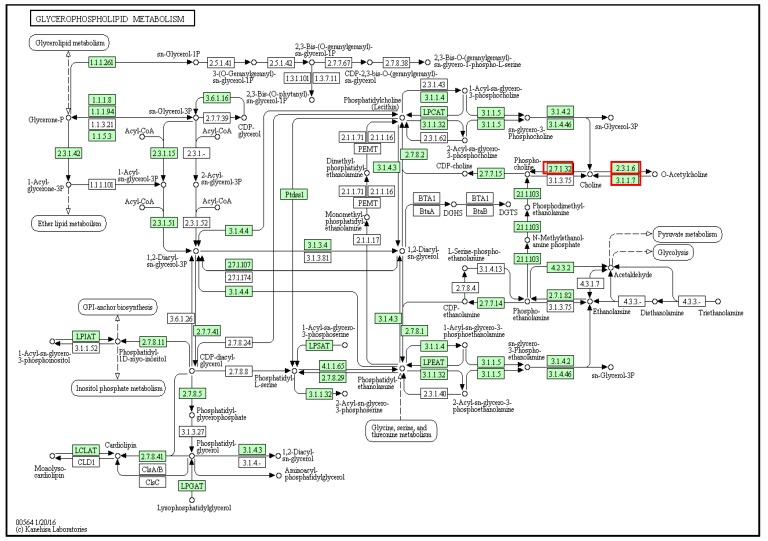
Glycerophospholipid metabolism pathway with matches to *D. orbita* contigs highlighted in green including choline kinase, choline *O*-acetyltransferase and acetylcholinesterase (**red** boxes) used to generate the acetyl choline moiety found in murexine.

Tyrindoxyl sulfate is stored as a choline ester of murexine, which contains a choline ester group and imidazole moiety (Figure 1B). The glycerophospholipid metabolism pathway has a role in the biosynthesis of choline esters [47] and more matches to reactions in the glycerophospholipid metabolism pathway (53 genes) were found in the *D. orbita* transcriptome compared to the annotated genomes of other molluscs (*L. gigantea* = 48, *O. bimaculoides* = 40 and *C. gigas* = 44 genes) (Figure S5). The *D. orbita* transcriptome included matches to choline/ethanol amine kinase (2.7.1.32; Figure 7), which produces choline from phosphocholine, as well as choline O-acetyltransferase (2.3.1.6; Figure 7) and acetylcholinesterase (3.1.1.7; Figure 7), which produce acetylcholine. Laffy et al. [33] found that acetylcholinesterase (3.1.1.7) transcripts were upregulated in the hypobranchial gland of *D. orbita* relative to foot tissue. The octopus *O. bimaculoides* and gastropod *L. gigantea* genomes were also found to contain matches to these enzymes for acetylcholine biosynthesis, whereas the oyster *C. gigas* lacks phosphocholine phosphatase. In comparison to the gastropods and the bivalve, the octopus *O. bimaculoides* was found to lack any matches to genes in the phosphothanolamine *N*-methyltransferase pathway for the production of phosphocholine from phosphoethanolamine.

**Figure 8:**
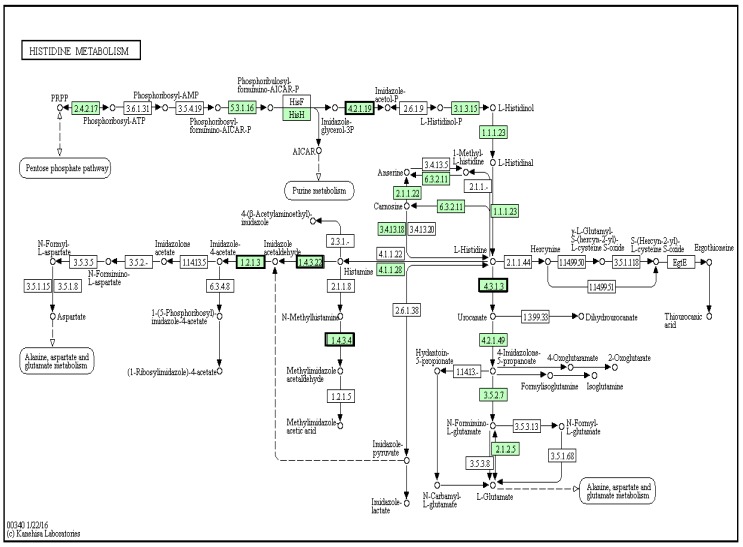
Histidine metabolism pathway showing matches to *Dicathais orbita* contigs highlighted in green, including several enzymes that convert histidine into imidazole (**red** boxes) and imidazoleglycerol-phosphate dehydratase (**red** box **top** pathway).

Choline or acetyl choline combines with imidazole to produce the muscle relaxant murexine [48] (Figure 1B). Imidazole is a derivative of the amino acid histidine [49], and there were several matches to the histidine metabolism pathway in the *D. orbita* transcriptome (Figure 8). These include, diamine oxidase (1.4.3.22; Figure 8), aldehyde dehydrogenase (NAD+) (1.2.1.3; Figure 8) and monoamine oxidase (1.4.3.4; Figure 8), which convert histamine into imidazole. There was also a match to histidine ammonia-lyase (4.3.1.3; Figure 8), which converts l-histidine to urocanate, which could feasibly combine with choline ester to form murexine. All these biosynthetic enzymes are also found in the *L. gigantea*, *O. bimaculoides* and *C. gigas* genomes.

Previous studies of choline esters in molluscs have focused on the predatory neogastropods, and there is no record of murexine or similar derivatives being isolated from limpets or oysters. Roseghini et al. [48] found no evidence for imidazole or acryl choline esters in 27 species from eight families of herbivorous and scavenging gastropods, including three Patellidae limpets, while at least one of these compounds was found in 53 of 55 species of the predatory Muricoidae superfamily. This implies the Neogastropoda have evolved a specific murexine biosynthesis pathway and, consistent with this, the *D. orbita* transcriptome had more matches to enzymes in the histidine metabolism pathway (19 genes) when compared to *L. gigantea* (10 genes), *O. bimaculoides* (11 genes) and *C. gigas* (13 genes) (Figure S6). Specifically, the enzyme involved in imidazole biosynthesis imidazoleglycerol-phosphate dehydratase (4.2.1.19; Figure 8) was only found in the *D. orbita* transcriptome. Overall, it appears the neogastropod *D. orbita* has evolved a complex suite of metabolic capabilities that are not represented in the more primitive orthogastropod or bivalve, for which complete genome sequences are available.

## 3. Materials and Methods

### 3.1. Specimen Collection

**Figure 9:**
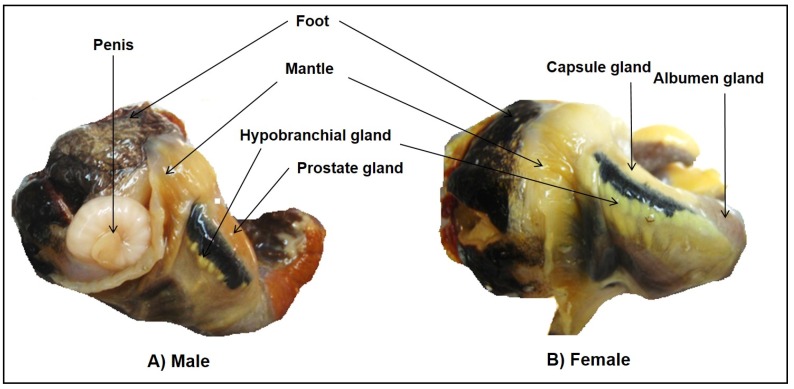
*Dicathais orbita* male (**A**) and female (**B**) tissues used for RNA extraction to generate the transcriptome.

Eighteen adult specimens of *D. orbita* (Table 1) were collected during low tide from the sub-tidal and intertidal rocky reefs of Flat rock, Ballina (28°84′ S and 153°60′ E), NSW, Australia. Six spawning females were collected during the breeding season August 2014 and a further six females and six males were collected after breeding season in January 2015, under the permit number F89/1171-6.0 issued by the Department of Primary Industries, NSW Government, Australia. Total RNA was extracted from the hypobranchial glands of the females collected in August 2014 and from five different tissues from female and three tissues from male *D. orbita* (Figure 9) collected in January 2015 (Table 1).

### 3.2. Transcriptome Sequencing

Three independent replicate snails were used for each tissue sample summarised in Table 1. The tissues were stabilized prior to RNA extraction in RNase free 2 ml Eppendorf tube using 600 μL of RNA*later* RNA stabilization reagent (Qiagen, Chadstone, Victoria, Australia). The stabilized tissue was incubated at 4 °C overnight and stored at −80 °C, prior to extracting the total RNA. The total RNA was extracted from the RNA*later* stabilized tissue using the E.Z.N.A. Mollusc RNA Kit (Omega Bio-tek, Norcross, GA, USA.) following the manufacturer’s instructions. The concentration and quality (purity and integrity) of total RNA was assessed by NanoDrop and the Agilent Bioanalyzer 2100 System (Agilent Technologies, Santa Clara, CA, USA). The total RNA extracted from three biological replicates of each tissue type (hypobranchial gland, prostate gland, albumen gland, capsule gland, mantle and foot) was pooled within the same tube for each tissue in equal masses. The pooled extracted RNA was stored at −80 °C until further used. The RNA samples were shipped to Macrogen Inc. (Seoul, Korea) for high throughput sequencing. Prior to shipping, each RNA sample was precipitated in a mixture of 2× ethanol (96%) and 0.1× sodium acetate (3 M). mRNA isolation and library construction were performed by Macrogen. The libraries were sequenced using the Illumina HiSeq 2000 platform (HCS2.2.38 version, Illumina, Seoul, Korea).

### 3.3. De Novo Transcriptome Assembly and Annotation

FASTQ format raw sequencing reads were checked for quality using FASTQC (version 0.10.4, http://www.bioinformatics.babraham.ac.uk/projects/fastqc) [34]. The adapter sequences, poly-N stretches and low quality reads (Phred score < 20) were removed using the BBDuck module of the BBMap software package (version 34_90, http://sourceforge.net/projects/bbmap) using command “bbduk.sh in1 = in1.fastq in2 = in2.fastq out1 = out1.fastq out2 = out2.fastq ref = adapters.fa qtrim = rl trimq = 20 ktrim = rl k = 28 hdist = 1 minlength = 20”. BLAST search of over-represented *k*-mers against NCBI confirmed these were mostly mitochondrial and ribosomal *Dicathais obita* genes, and no evidence of bacterial contamination was found. CLC Genomics Workbench, version 4.9 (CLC Bio, Aarhus, Denmark; www.clcbio.com) with the option to map reads back to contigs, automatic word size and automatic bubble size was used to de novo assemble the high quality reads. All the contigs were clustered using CD-hit-est (version v4.6.1, http://weizhongli-lab.org/cd-hit) [50], and Transdecoder (version 2.0.3, http://transdecoder.github.io/) was used to identify candidate coding regions within transcript sequences.

BLAST analysis was done against non-redundant protein database Nr (Mollusc specific proteins), Swiss-Prot and KEGG protein databases. Gene ontology analysis was performed with Interproscan (version 5.10.50, https://code.google.com/archive/p/interproscan) [51] using command “interproscan.sh -appl ProDom,PfamA,PANTHER -i longest_orfs.pep.fa -o out.txt -f TSV -goterms -iprlookup -pa” and plotted using WEGO (http://wego.genomics.org.cn/cgi-bin/wego/index.p) [52]. *D. orbita* transcripts were searched for bromoperoxidase gene by aligning against known bromoperoxidase genes available in the NCBI GenBank using BLAST (e value 1 × 10^−3^) (Supplementary Table S3).

### 3.4. Nucleotide Sequence Accession Number

All raw sequence data were deposited in the European nucleotide archive (ENA) with the accession numbers PRJEB12262. Assembled contigs are available from the authors upon request.

## 4. Conclusions

This transcriptome study of *D. orbita* generated over 216 million high quality reads that were de novo assembled into 219,437 contigs, of which 76,152 contigs contained candidate-coding regions that were annotated with Nr, Swiss-Prot and KEGG databases. This provides a significant new molecular resource for neogastropod molluscs, and adds to pool of genomic data for molluscs in general. Several genes that are potentially associated with Tyrian purple precursor biosynthesis in *D. orbita* were identified. It appears the neogastropod *D. orbita* has evolved a complex suite of metabolic capabilities that are not represented in the more primitive orthogastropods or bivalves, for which complete genome sequences are available.

## Figures and Tables

**Table 1:** Summary of the number of raw sequencing reads and the percent remaining after quality control from 14 tissue samples of *Dicathais orbita*.

Snails (S)	Description	Raw Reads	High Quality Reads	
			Number	Percent
S1 + S2 + S3	Female hypobranchial gland 1, August, Breeding season, 2014	15,531,322	15,100,466	97.23
S4 + S5 + S6	Female hypobranchial gland 2, August, Breeding season, 2014	15,693,385	15,258,671	97.23
S7 + S8 + S9	Female hypobranchial gland 1, January, 2015	15,835,271	15,425,533	97.41
S10 + S11 + S12	Female hypobranchial gland 2, January, 2015	16,457,635	15,990,724	97.16
S13 + S14 + S15	Male hypobranchial gland 1, January, 2015	16,142,317	15,684,926	97.17
S16 + S17 + S18	Male hypobranchial gland 2, January, 2015	17,461,007	16,997,497	97.35
S7 + S8 + S9	Female foot 1, January, 2015	16,015,535	15,595,463	97.38
S10 + S11 + S12	Female foot 2, January, 2015	17,057,433	16,653,222	91.40
S13 + S14 + S15	Male foot 1, January, 2015	14,241,690	13,885,327	97.50
S16 + S17 + S18	Male foot 2, January, 2015	15,813,363	15,406,030	97.42
S7 + S8 + S9	Capsule gland, January, 2015	15,805,867	15,291,498	96.75
S7 + S8 + S9	Albumen gland, January, 2015	14,442,864	14,011,099	97.01
S13 + S14 + S15	Prostate gland, January, 2015	15,600,688	15,113,842	96.88
S10 + S11 + S12	Mantle 1, January, 2015	16,273,556	15,804,247	97.12
-	Total	222,371,933	216,218,545	-

**Table 2:** Summary statistics of the assembled contigs using CLC Genomics de novo assembler.

Contig Summary Statistics	bp (Base Pair)
Number of contigs	219,437
Total assembly length	117,767,308
N50	608
Mean contig length	537
Largest contig length	12,897
Number of contigs larger than 500 bp	59,144
Number of contigs larger than 1000 bp	22,818

## Supplementary Materials

The following are available online at www.mdpi.com/1660-3397/14/7/135/s1, Figure S1: Tryptophan metabolism pathways for (A) *Crassostrea gigas*; (B) *Lottia gigantean*; and (C) *Octopus bimaculoides* showing enzyme matches in green including tryptophan 5-monoxygenase (red box), which was missing form *Dicathais orbita*, but no match to tryptophanase (blue box), Figure S2: Phenylalanine, tyrosine and tryptophan biosynthetic pathways for (A) *Crassostrea gigas*; (B) *Lottia gigantean*; and (C) *Octopus bimaculoides* with enzyme matches in green, but with no match to with tryptophan synthase highlighted in the blue box, Figure S3: Sulfur metabolism pathways for (A) *Crassostrea gigas*; (B) *Lottia gigantea*; and (C) *Octopus bimaculoides*, Figure S4: Cysteine and methionine metabolism pathway of (A) *Crassostrea gigas*; (B) *Lottia gigantea*; and (C) *Octopus bimaculoides*, Figure S5: Glycerophospholipid metabolism pathway of (A) *Crassostrea gigas*; (B) *Lottia gigantea*; and (C) *Octopus bimaculoides* showing enzyme matches in green with those relevant to choline ester synthesis highlighted in red (present) and blue (absent), Figure S6: Histidine metabolism pathway of (A) *Crassostrea gigas*; (B) *Lottia gigantea*; and (C) *Octopus bimaculoides* showing matching enzymes in green, including several enzymes that convert histidine into imidazole (red boxes) but not imidazoleglycerol-phosphate dehydratase (blue box), Table S1: Analysis of KEGG pathway showing the top 20 metabolic pathway involving the largest number of contigs in *D. orbita* trancriptome, Table S2: List of the 28 mapped contigs and with the KEGG orthology assignment in *D. orbita* trancriptome for tryptophan metabolism, Table S3: List of known bromoperoxidsae genes available in NCBI GenBank used for BLAST against *D. orbita*.

## Abbreviations

The following abbreviations are used in this manuscript:

ENA	European Nucleotide Archive
NCBI	National Center for Biotechnology Information
BLAST	Basic Local Alignment Search Tool
ORFs	Open Reading Frames
KEGG	Kyoto Encyclopedia of Genes and Genomes
