# Supplementary material for: Transcriptome of the Australian Mollusc Dicathais orbita Provides Insights into the Biosynthesis of Indoles and Choline Esters
Source: Mar Drugs. 2016 Jul 20;14(7):135. doi: 10.3390/md14070135 (PMC4962025; doi:10.3390/md14070135)
Supplement: Supplementary file 1 [file marinedrugs-14-00135-s001.pdf]

Supplementary Material: Transcriptome of the Australian Mollusc *Dicathais orbita* Provides Insights into the Biosynthesis of Indoles and Choline Esters

Abdul Baten, Ajit Kumar Ngangbam, Daniel L. E. Waters and Kirsten Benkendorff

Table S1. Analysis of KEGG pathway showing the top 20 metabolic pathway involving the largest number of contigs in *D. orbita* trancriptome.

Sl. No.	Top 20 Metabolic Pathway
1	ko01100 Metabolic pathways (914)
2	ko01110 Biosynthesis of secondary metabolites (304)
3	ko01130 Biosynthesis of antibiotics (211)
4	ko01120 Microbial metabolism in diverse environments (173)
5	ko00230 Purine metabolism (120)
6	ko03013 RNA transport (118)
7	ko04144 Endocytosis (1118)
8	ko03010 Ribosome (117)
9	ko01200 Carbon metabolism (113)
10	ko05016 Huntington's disease (112)
11	ko03040 Spliceosome (106)
12	ko00190 Oxidative phosphorylation (105)
13	ko04141 Protein processing in endoplasmic reticulum (103)
14	ko00240 Pyrimidine metabolism (98)
15	ko05010 Alzheimer's disease (92)
16	ko01230 Biosynthesis of amino acids (91)
17	ko04120 Ubiquitin mediated proteolysis (88)
18	ko05169 Epstein-Barr virus infection (88)
19	ko05166 HTLV-I infection (86)
20	ko04142 Lysosome (80)

Table S2. List of the 28 mapped contigs and with the KEGG orthology assignment in *D. orbita* trancriptome for tryptophan metabolism.

Sl. No.	List of the Enzymes in *D. orbita* Trancriptome for Tryptophan Metabolism
1	ko:K00022 HADH; 3-hydroxyacyl-CoA dehydrogenase [EC:1.1.1.35]
2	ko:K00128 E1.2.1.3; aldehyde dehydrogenase (NAD+) [EC:1.2.1.3]
3	ko:K00149 ALDH9A1; aldehyde dehydrogenase family 9 member A1 [EC:1.2.1.47 1.2.1.3]
4	ko:K00164 OGDH; 2-oxoglutarate dehydrogenase E1 component [EC:1.2.4.2]
5	ko:K00252 GCDH; glutaryl-CoA dehydrogenase [EC:1.3.8.6]
6	ko:K00274 MAO; monoamine oxidase [EC:1.4.3.4]
7	ko:K00452 HAAO; 3-hydroxyanthranilate 3,4-dioxygenase [EC:1.13.11.6]
8	ko:K00453 E1.13.11.11; tryptophan 2,3-dioxygenase [EC:1.13.11.11]
9	ko:K00463 INDO; indoleamine 2,3-dioxygenase [EC:1.13.11.52]
10	ko:K00486 KMO; kynurenine 3-monooxygenase [EC:1.14.13.9]
11	ko:K00626 E2.3.1.9; acetyl-CoA C-acetyltransferase [EC:2.3.1.9]
12	ko:K00816 CCBL; kynurenine—oxoglutarate transaminase/cysteine-S-conjugate beta-lyase/glutamine—phenylpyruvate transaminase [EC:2.6.1.7 4.4.1.13 2.6.1.64]
13	ko:K00825 AADAT; kynurenine/2-aminoadipate aminotransferase [EC:2.6.1.7 2.6.1.39]
14	ko:K01426 E3.5.1.4; amidase [EC:3.5.1.4]
15	ko:K01432 AFMID; arylformamidase [EC:3.5.1.9]
16	ko:K01556 KYNU; kynureninase [EC:3.7.1.3]
17	ko:K01593 DDC; aromatic-L-amino-acid decarboxylase [EC:4.1.1.28]
18	ko:K01667 tnaA; tryptophanase [EC:4.1.99.1]
19	ko:K01692 paaF; enoyl-CoA hydratase [EC:4.2.1.17]
20	ko:K01782 fadJ; 3-hydroxyacyl-CoA dehydrogenase/enoyl-CoA hydratase/3-hydroxybutyryl-CoA epimerase [EC:1.1.1.35 4.2.1.17 5.1.2.3]
21	ko:K03781 katE; catalase [EC:1.11.1.6]
22	ko:K07408 CYP1A1; cytochrome P450, family 1, subfamily A, polypeptide 1 [EC:1.14.14.1]
23	ko:K07511 ECHS1; enoyl-CoA hydratase [EC:4.2.1.17]
24	ko:K07514 EHHADH; enoyl-CoA hydratase/3-hydroxyacyl-CoA dehydrogenase/3,2-trans-enoyl-CoA isomerase [EC:4.2.1.17 1.1.1.35 5.3.3.8]
25	ko:K07515 HADHA; enoyl-CoA hydratase/long-chain 3-hydroxyacyl-CoA dehydrogenase [EC:4.2.1.17 1.1.1.211]
26	ko:K11182 AOC1; diamine oxidase [EC:1.4.3.22]
27	ko:K11818 CYP83B1; cytochrome P450, family 83, subfamily B, polypeptide 1 [EC:1.14.-.-]
28	ko:K14085 ALDH7A1; aldehyde dehydrogenase family 7 member A1 [EC:1.2.1.31 1.2.1.8 1.2.1.3]

Table S3. List of known bromoperoxidsae genes available in NCBI GenBank used for BLAST against *D. orbita*.

Accession Numbers	Source Species
AJ491786.1	*Laminaria digitata*
JX128092.1	*Gracilaria changii*
AF218810.1	*Corallina officinalis*
XM_001822243.2	*Aspergillus oryzae*
XM_003234475.1	*Trichophyton rubrum*
XM_002679630.1	*Naegleria gruberi*
NM_001157259.1	*Zea mays*
KLO55315.1	*Mycobacterium haemophilum*
AKJ56790.1	*Bacillus thuringiensis*
KLI98717.1	*Luteimonas* sp. FCS-9
YP_029199.1	*Bacillus anthracis* str. Sterne
KKM37545.1	*Bacillus anthracis*
KKI91829.1	*Bacillus* sp. SA1-12
KXH89531	*Bacillus* sp. JH7
KXI43580	*Bacillus cereus*
ALQ29400.1	*Arthrobacter* sp. YC-RL1
KND40451.1	*Streptomyces stelliscabiei*
WP_046733028	*Streptomyces* sp. MUSC119T

A

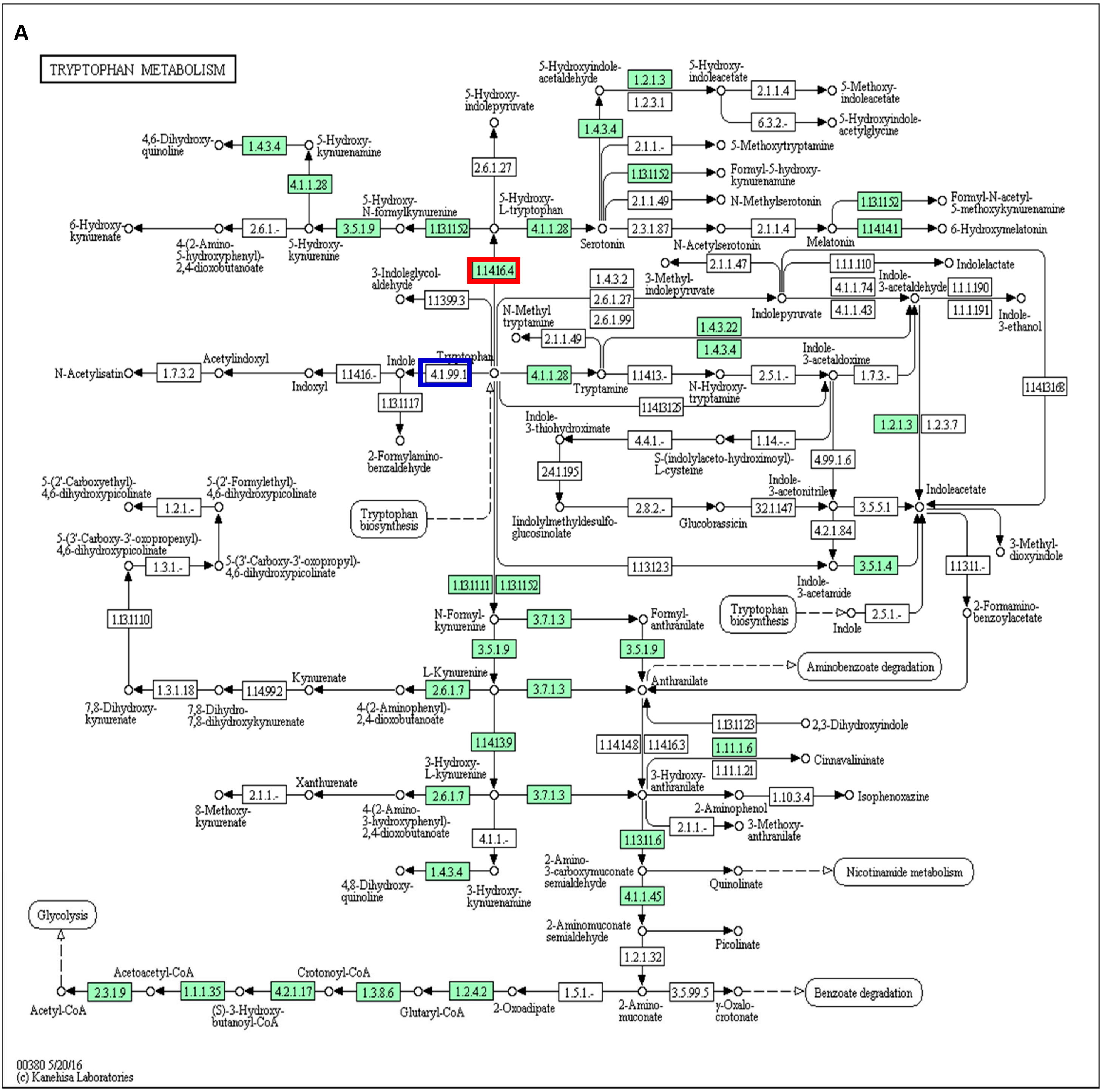
TRYPTOPHAN METABOLISM
Tryptophan
Indole
Serotonin
Melatonin
Tryptamine
L-Kynurenine
N-Formyl-kynurenine
Kynurenate
Anthranilate
3-Hydroxy-L-kynurenine
3-Hydroxy-anthranilate
Xanthurenate
Quinolinate
Picolinate
Indoleacetate
Indolepyruvate
Acetyl-CoA
Glutaryl-CoA
2-Oxoadipate
Tryptophan biosynthesis
Glycolysis
Nicotinamide metabolism
Benzoate degradation
Aminobenzoate degradation
1.14.16.4
4.1.99.1
00380 5/20/16
(c) Kanehisa Laboratories

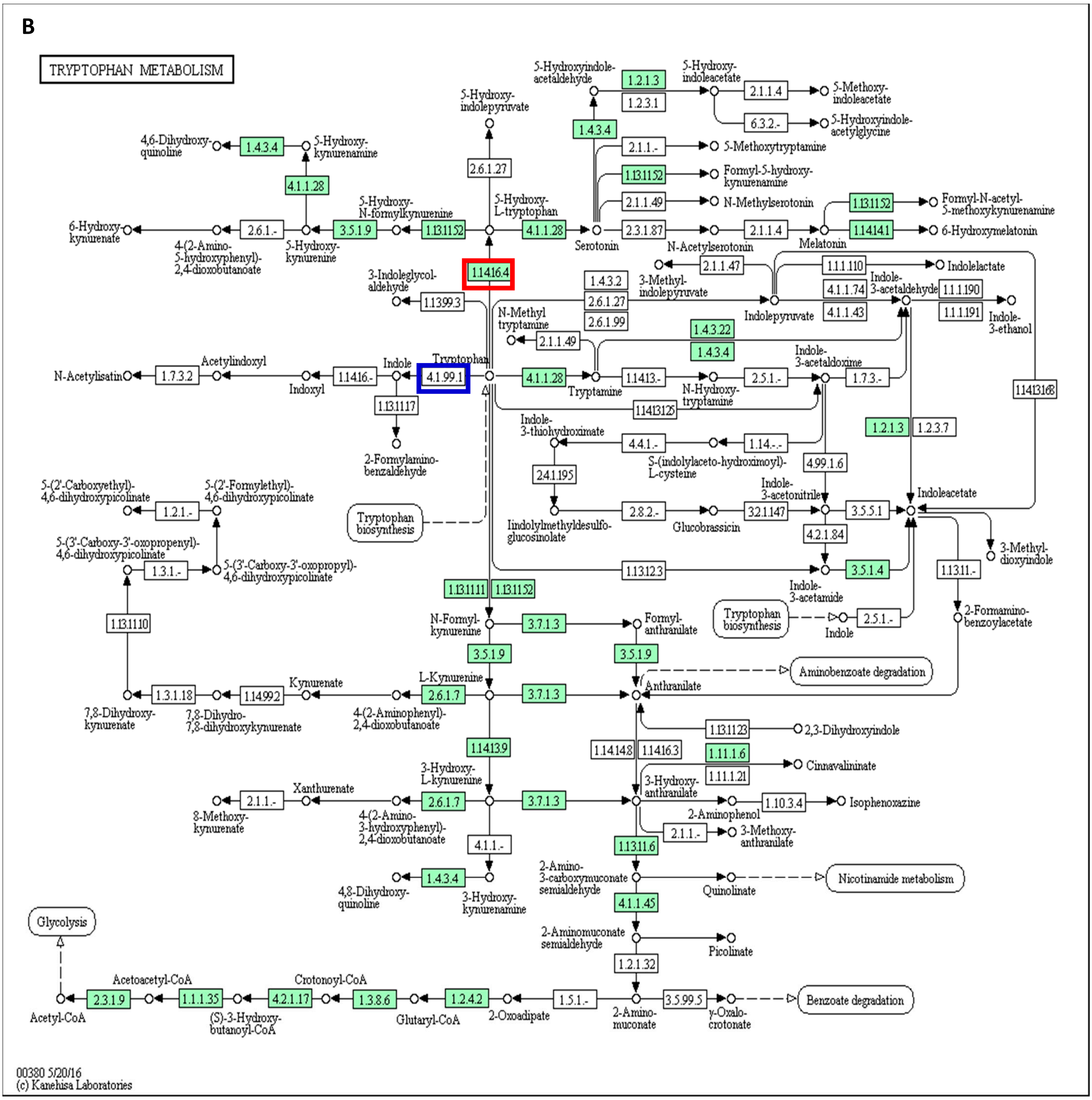
B
TRYPTOPHAN METABOLISM
5-Hydroxyindole-acetaldehyde
1.2.1.3
1.2.3.1
5-Hydroxy-indoleacetate
2.1.1.4
5-Methoxy-indoleacetate
6.3.2.-
5-Hydroxyindole-acetylglycine
5-Hydroxy-indolepyruvate
1.4.3.4
2.1.1.-
5-Methoxytryptamine
1.13.11.52
Formyl-5-hydroxy-kynurenamine
2.1.1.49
N-Methylserotonin
4,6-Dihydroxy-quinoline
1.4.3.4
5-Hydroxy-kynurenamine
4.1.1.28
2.6.1.27
5-Hydroxy-N-formylkynurenine
5-Hydroxy-L-tryptophan
6-Hydroxy-kynurenate
2.6.1.-
3.5.1.9
1.13.11.52
4.1.1.28
Serotonin
2.3.1.87
2.1.1.4
Melatonin
1.13.11.52
Formyl-N-acetyl-5-methoxykynurenamine
1.14.14.1
6-Hydroxymelatonin
4-(2-Amino-5-hydroxyphenyl)-2,4-dioxobutanoate
5-Hydroxy-kynurenine
N-Acetylserotonin
2.1.1.47
1.1.1.110
Indolelactate
1.14.16.4
3-Indoleglycol-aldehyde
1.13.99.3
1.4.3.2
2.6.1.27
2.6.1.99
3-Methyl-indolepyruvate
Indolepyruvate
4.1.1.74
4.1.1.43
Indole-3-acetaldehyde
1.1.1.190
1.1.1.191
Indole-3-ethanol
N-Methyl tryptamine
2.1.1.49
1.4.3.22
1.4.3.4
N-Acetylisatin
1.7.3.2
Acetylindoxyl
Indoxyl
1.14.16.-
Indole
4.1.99.1
Tryptophan
4.1.1.28
Tryptamine
1.14.13.-
N-Hydroxy-tryptamine
2.5.1.-
Indole-3-acetaldoxime
1.7.3.-
1.14.13.168
1.13.11.17
1.14.13.125
2-Formylamino-benzaldehyde
Indole-3-thiohydroximate
4.4.1.-
1.14.-.-
S-(indolylacetо-hydroximoyl)-L-cysteine
4.99.1.6
1.2.1.3
1.2.3.7
2.4.1.195
Indole-3-acetonitrile
Indoleacetate
5-(2'-Carboxyethyl)-4,6-dihydroxypicolinate
5-(2'-Formylethyl)-4,6-dihydroxypicolinate
1.2.1.-
Tryptophan biosynthesis
2.8.2.-
3.2.1.147
3.5.5.1
Indolylmethyl-desulfo-glucosinolate
Glucobrassicin
4.2.1.84
3-Methyl-dioxyindole
5-(3'-Carboxy-3'-oxopropenyl)-4,6-dihydroxypicolinate
1.3.1.-
5-(3'-Carboxy-3'-oxopropyl)-4,6-dihydroxypicolinate
1.13.12.3
3.5.1.4
1.13.11.-
1.13.11.11
1.13.11.52
Indole-3-acetamide
1.13.11.10
N-Formyl-kynurenine
3.7.1.3
Formyl-anthranilate
Tryptophan biosynthesis
Indole
2.5.1.-
2-Formamino-benzoylacetate
3.5.1.9
3.5.1.9
Aminobenzoate degradation
L-Kynurenine
Kynurenate
Anthranilate
7,8-Dihydroxy-kynurenate
1.3.1.18
7,8-Dihydro-7,8-dihydroxykynurenate
1.14.99.2
2.6.1.7
3.7.1.3
4-(2-Aminophenyl)-2,4-dioxobutanoate
1.13.11.23
2,3-Dihydroxyindole
1.14.13.9
1.14.14.8
1.14.16.3
1.11.1.6
Cinnavalininate
3-Hydroxy-L-kynurenine
1.11.1.21
Xanthurenate
3-Hydroxy-anthranilate
8-Methoxy-kynurenate
2.1.1.-
2.6.1.7
3.7.1.3
2-Aminophenol
1.10.3.4
Isophenoxazine
4-(2-Amino-3-hydroxyphenyl)-2,4-dioxobutanoate
4.1.1.-
2.1.1.-
3-Methoxy-anthranilate
1.13.11.6
1.4.3.4
2-Amino-3-carboxymuconate semialdehyde
Quinolinate
Nicotinamide metabolism
4,8-Dihydroxy-quinoline
3-Hydroxy-kynurenamine
4.1.1.45
Glycolysis
2-Aminomuconate semialdehyde
Picolinate
1.2.1.32
Acetoacetyl-CoA
Crotonoyl-CoA
2.3.1.9
1.1.1.35
4.2.1.17
1.3.8.6
1.2.4.2
1.5.1.-
3.5.99.5
Benzoate degradation
Acetyl-CoA
(S)-3-Hydroxy-butanoyl-CoA
Glutaryl-CoA
2-Oxoadipate
2-Amino-muconate
γ-Oxalo-crotonate
00380 5/20/16
(c) Kanehisa Laboratories

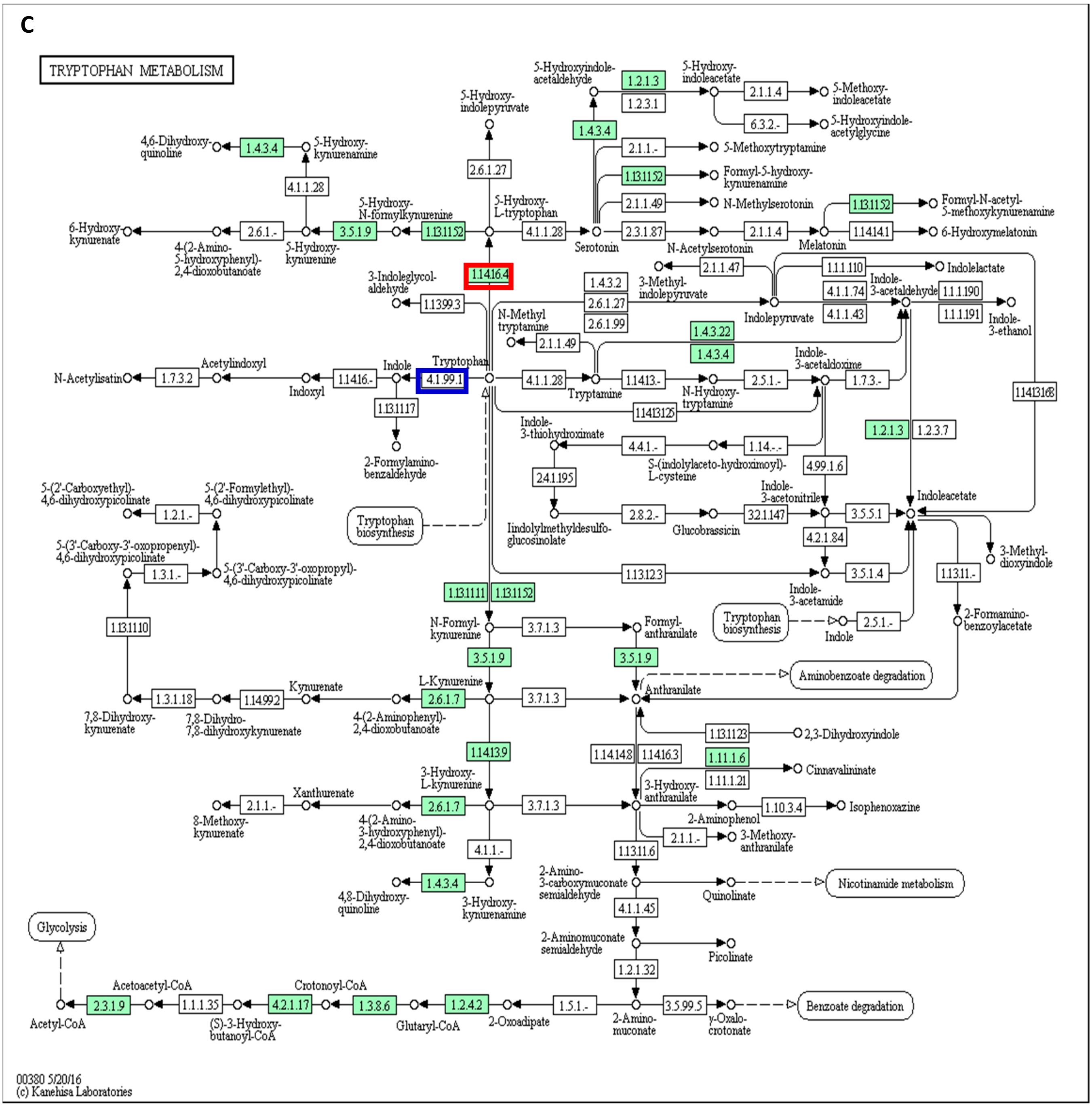

Figure S1. Tryptophan metabolism pathways for (**A**) *Crassostrea gigas*, (**B**) *Lottia gigantea* and, (**C**) *Octopus bimaculoides* showing enzyme matches in green including tryptophan 5-monoxygenase (red box), which was missing form *Dicathais orbita*, but no match to tryptophanase (blue box).

A

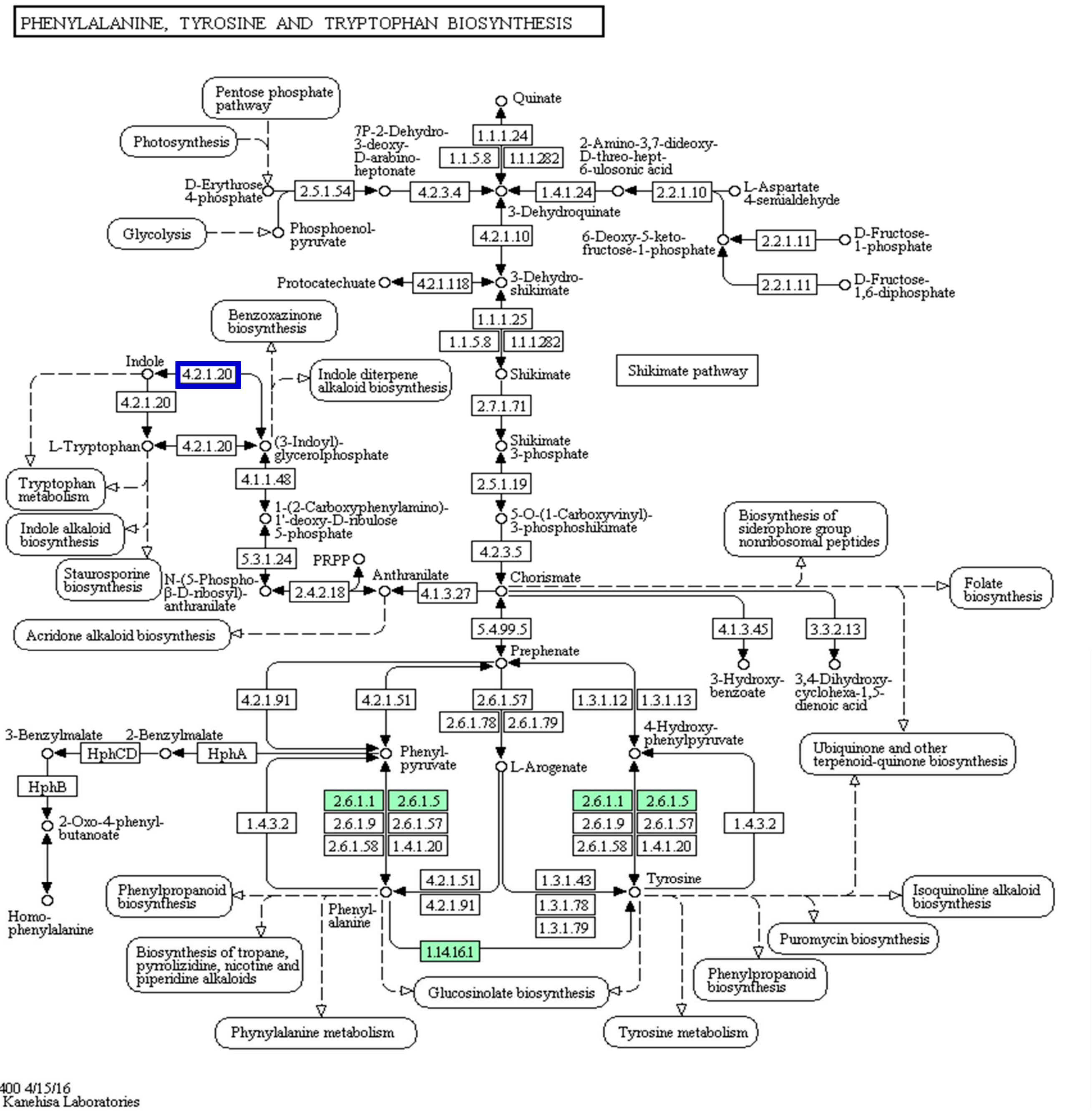
PHENYLALANINE, TYROSINE AND TRYPTOPHAN BIOSYNTHESIS
Pentose phosphate pathway
Photosynthesis
Glycolysis
D-Erythrose 4-phosphate
Phosphoenol-pyruvate
2.5.1.54
4.2.3.4
7P-2-Dehydro-3-deoxy-D-arabino-heptonate
Quinate
1.1.1.24
1.1.5.8
1.1.1.282
2-Amino-3,7-dideoxy-D-threo-hept-6-ulosonic acid
1.4.1.24
2.2.1.10
L-Aspartate 4-semialdehyde
3-Dehydroquinate
4.2.1.10
6-Deoxy-5-keto-fructose-1-phosphate
2.2.1.11
D-Fructose-1-phosphate
D-Fructose-1,6-diphosphate
Protocatechuate
4.2.1.118
3-Dehydro-shikimate
1.1.1.25
Benzoxazinone biosynthesis
Indole
4.2.1.20
Indole diterpene alkaloid biosynthesis
Shikimate
Shikimate pathway
2.7.1.71
L-Tryptophan
(3-Indoyl)-glycerolphosphate
Shikimate 3-phosphate
Tryptophan metabolism
4.1.1.48
2.5.1.19
1-(2-Carboxyphenylamino)-1'-deoxy-D-ribulose 5-phosphate
Indole alkaloid biosynthesis
5-O-(1-Carboxyvinyl)-3-phosphoshikimate
Biosynthesis of siderophore group nonribosomal peptides
5.3.1.24
PRPP
4.2.3.5
Staurosporine biosynthesis
N-(5-Phospho-β-D-ribosyl)-anthranilate
2.4.2.18
Anthranilate
4.1.3.27
Chorismate
Folate biosynthesis
Acridone alkaloid biosynthesis
5.4.99.5
4.1.3.45
3.3.2.13
Prephenate
3-Hydroxy-benzoate
3,4-Dihydroxy-cyclohexa-1,5-dienoic acid
4.2.1.91
4.2.1.51
2.6.1.57
1.3.1.12
1.3.1.13
2.6.1.78
2.6.1.79
3-Benzylmalate
2-Benzylmalate
HphCD
HphA
Phenyl-pyruvate
4-Hydroxy-phenylpyruvate
L-Arogenate
Ubiquinone and other terpenoid-quinone biosynthesis
HphB
2.6.1.1
2.6.1.5
2-Oxo-4-phenyl-butanoate
1.4.3.2
2.6.1.9
2.6.1.58
1.4.1.20
Homo-phenylalanine
Phenylpropanoid biosynthesis
4.2.1.51
4.2.1.91
1.3.1.43
1.3.1.78
1.3.1.79
Tyrosine
Isoquinoline alkaloid biosynthesis
Phenyl-alanine
Puromycin biosynthesis
Biosynthesis of tropane, pyrrolizidine, nicotine and piperidine alkaloids
1.14.16.1
Phenylpropanoid biosynthesis
Glucosinolate biosynthesis
Phynylalanine metabolism
Tyrosine metabolism
00400 4/15/16
(c) Kanehisa Laboratories

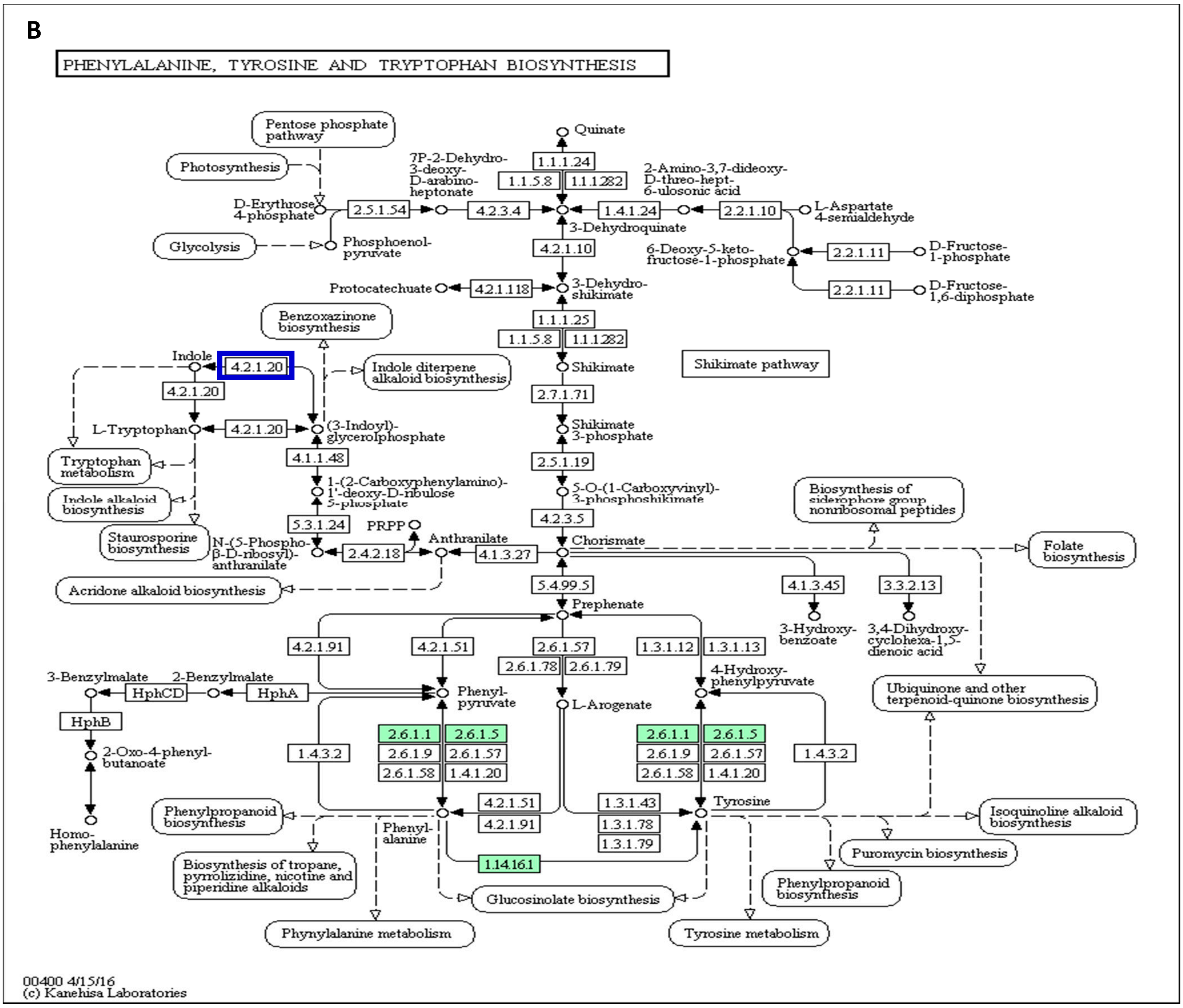
B
PHENYLALANINE, TYROSINE AND TRYPTOPHAN BIOSYNTHESIS
Pentose phosphate pathway
Photosynthesis
Glycolysis
D-Erythrose 4-phosphate
Phosphoenol-pyruvate
2.5.1.54
7P-2-Dehydro-3-deoxy-D-arabino-heptonate
4.2.3.4
Quinate
1.1.1.24
1.1.5.8
1.1.1.282
2-Amino-3,7-dideoxy-D-threo-hept-6-ulosonic acid
1.4.1.24
2.2.1.10
L-Aspartate 4-semialdehyde
3-Dehydroquinate
4.2.1.10
6-Deoxy-5-keto-fructose-1-phosphate
2.2.1.11
D-Fructose-1-phosphate
D-Fructose-1,6-diphosphate
Protocatechuate
4.2.1.118
3-Dehydro-shikimate
1.1.1.25
Shikimate
2.7.1.71
Shikimate 3-phosphate
2.5.1.19
5-O-(1-Carboxyvinyl)-3-phosphoshikimate
4.2.3.5
Chorismate
Shikimate pathway
Benzoxazinone biosynthesis
Indole diterpene alkaloid biosynthesis
Indole
4.2.1.20
L-Tryptophan
(3-Indoyl)-glycerolphosphate
4.1.1.48
1-(2-Carboxyphenylamino)-1'-deoxy-D-ribulose 5-phosphate
5.3.1.24
PRPP
Anthranilate
4.1.3.27
2.4.2.18
N-(5-Phospho-β-D-ribosyl)-anthranilate
Tryptophan metabolism
Indole alkaloid biosynthesis
Staurosporine biosynthesis
Acridone alkaloid biosynthesis
Biosynthesis of siderophore group nonribosomal peptides
Folate biosynthesis
4.1.3.45
3.3.2.13
3-Hydroxy-benzoate
3,4-Dihydroxy-cyclohexa-1,5-dienoic acid
5.4.99.5
Prephenate
4.2.1.91
4.2.1.51
2.6.1.57
2.6.1.78
2.6.1.79
1.3.1.12
1.3.1.13
Phenyl-pyruvate
L-Arogenate
4-Hydroxy-phenylpyruvate
Ubiquinone and other terpenoid-quinone biosynthesis
3-Benzylmalate
2-Benzylmalate
HphCD
HphA
HphB
2-Oxo-4-phenyl-butanoate
Homo-phenylalanine
2.6.1.1
2.6.1.5
2.6.1.9
2.6.1.58
1.4.1.20
1.4.3.2
1.3.1.43
1.3.1.78
1.3.1.79
1.14.16.1
Phenylalanine
Tyrosine
Phenylpropanoid biosynthesis
Biosynthesis of tropane, pyrrolizidine, nicotine and piperidine alkaloids
Glucosinolate biosynthesis
Phynylalanine metabolism
Tyrosine metabolism
Isoquinoline alkaloid biosynthesis
Puromycin biosynthesis
00400 4/15/16
(c) Kanehisa Laboratories

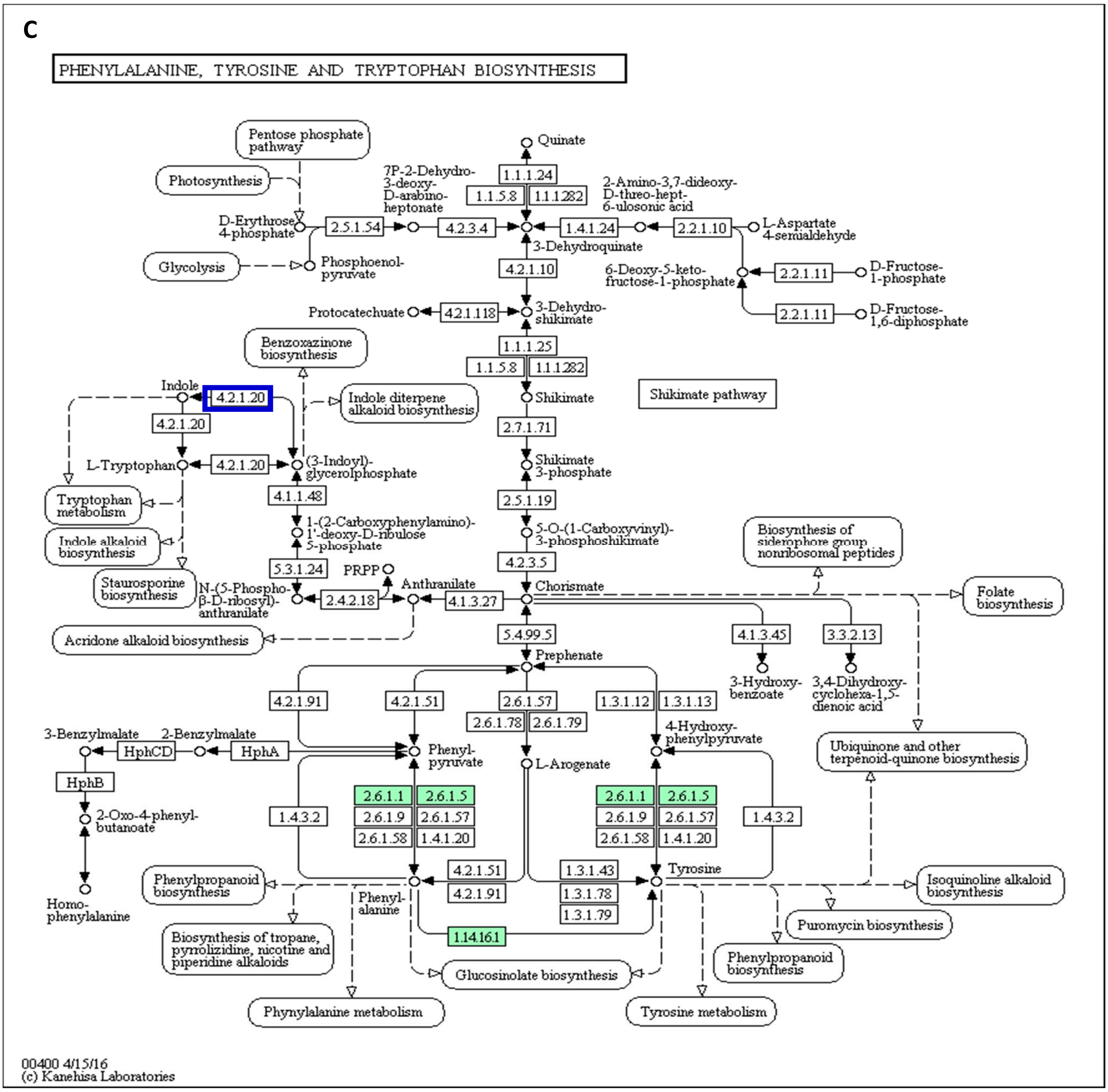

Figure S2. Phenylalanine, tyrosine and tryptophan biosynthetic pathways for (**A**) *Crassostrea gigas*, (**B**) *Lottia gigantea* and, (**C**) *Octopus bimaculoides* with enzyme matches in green, but with no match to tryptophan synthase highlighted in the blue box.

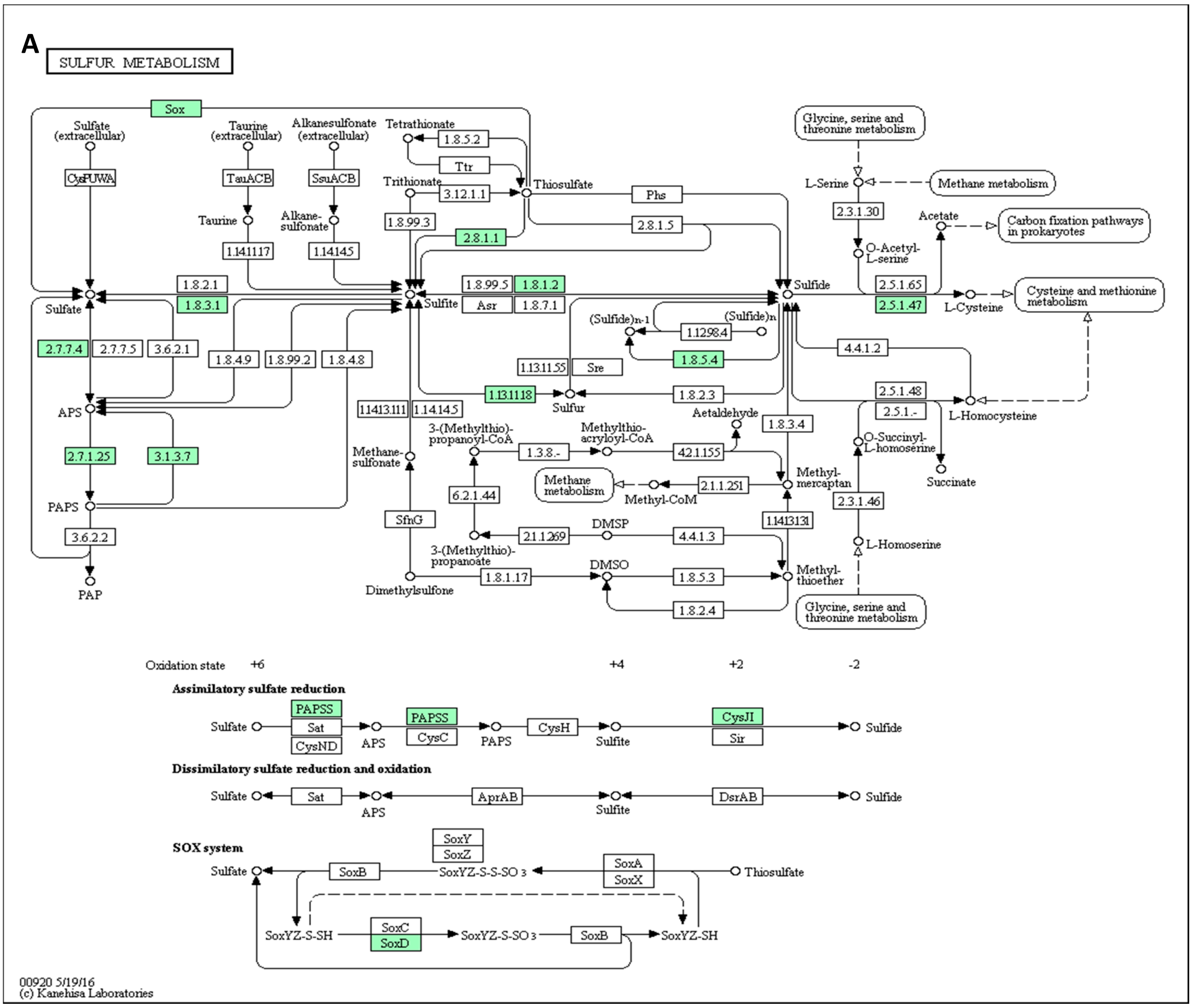
A
SULFUR METABOLISM
Sox
Sulfate (extracellular)
Taurine (extracellular)
Alkanesulfonate (extracellular)
Tetrathionate
CysPUWA
TauACB
SsuACB
Taurine
Alkane-sulfonate
Trithionate
Thiosulfate
Sulfite
Sulfide
Sulfate
APS
PAPS
PAP
Sulfur
Methane-sulfonate
Dimethylsulfone
DMSP
DMSO
Methyl-mercaptan
Methyl-thioether
Methyl-CoM
Methane metabolism
L-Serine
O-Acetyl-L-serine
Acetate
L-Cysteine
L-Homocysteine
O-Succinyl-L-homoserine
L-Homoserine
Succinate
Glycine, serine and threonine metabolism
Carbon fixation pathways in prokaryotes
Cysteine and methionine metabolism
Oxidation state +6 +4 +2 -2
Assimilatory sulfate reduction
PAPSS
Sat
CysND
CysC
CysH
CysJI
Sir
Dissimilatory sulfate reduction and oxidation
AprAB
DsrAB
SOX system
SoxY
SoxZ
SoxA
SoxX
SoxB
SoxC
SoxD
SoxYZ-S-SH
SoxYZ-SH
00920 5/19/16
(c) Kanehisa Laboratories

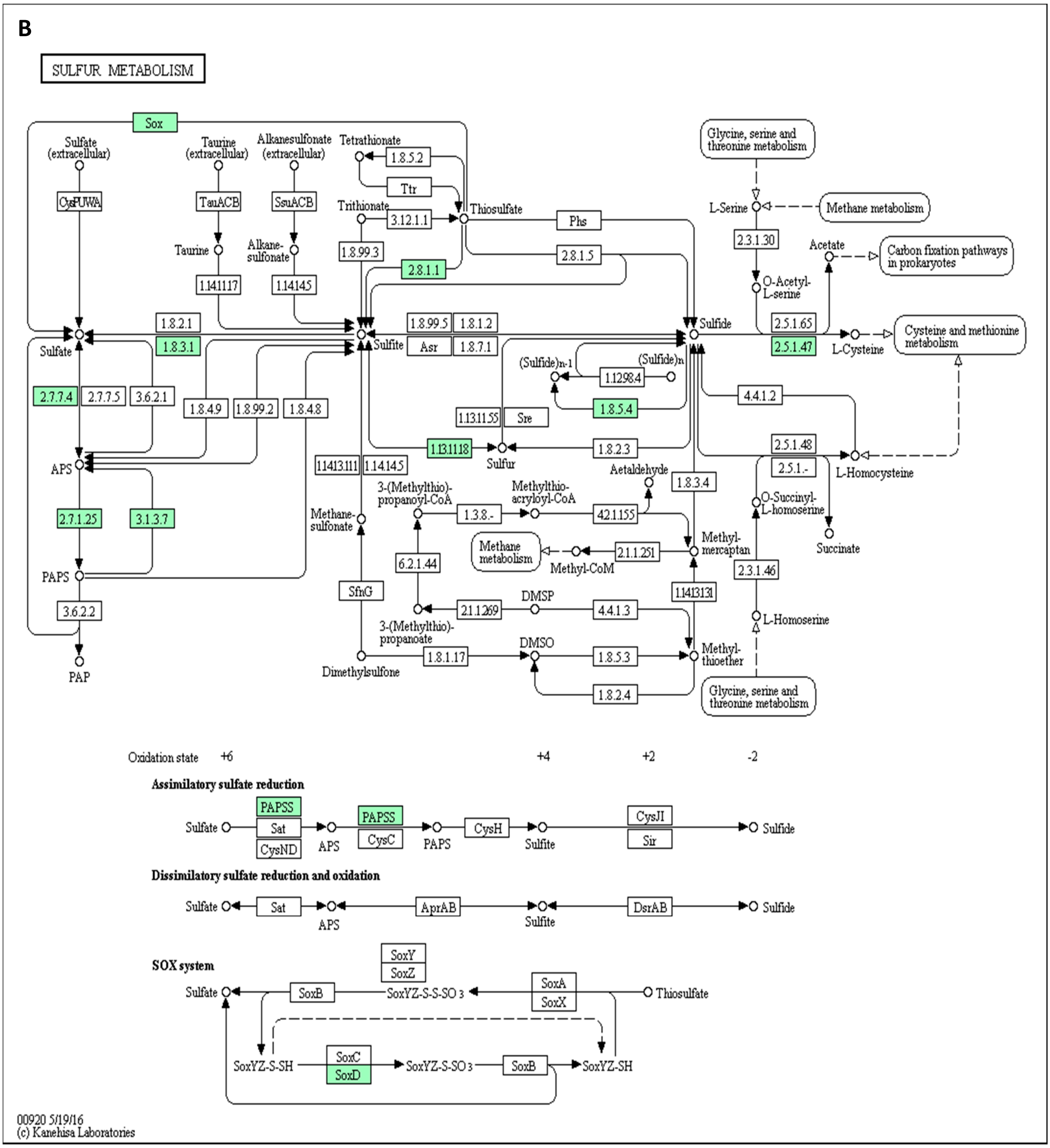
B
SULFUR METABOLISM
Sox
Sulfate (extracellular)
Taurine (extracellular)
Alkanesulfonate (extracellular)
Tetrathionate
Trithionate
Thiosulfate
Sulfite
Sulfide
Sulfate
APS
PAPS
PAP
Sulfur
DMSP
DMSO
Dimethylsulfone
Methanesulfonate
Methyl-mercaptan
Methyl-thioether
Methyl-CoM
L-Serine
O-Acetyl-L-serine
L-Cysteine
L-Homocysteine
O-Succinyl-L-homoserine
L-Homoserine
Succinate
Acetate
Aetaldehyde
Glycine, serine and threonine metabolism
Methane metabolism
Carbon fixation pathways in prokaryotes
Cysteine and methionine metabolism
Oxidation state +6 +4 +2 -2
Assimilatory sulfate reduction
PAPSS
Sat
CysND
CysC
CysH
CysJI
Sir
Dissimilatory sulfate reduction and oxidation
AprAB
DsrAB
SOX system
SoxB
SoxY
SoxZ
SoxA
SoxX
SoxC
SoxD
00920 5/19/16
(c) Kanehisa Laboratories

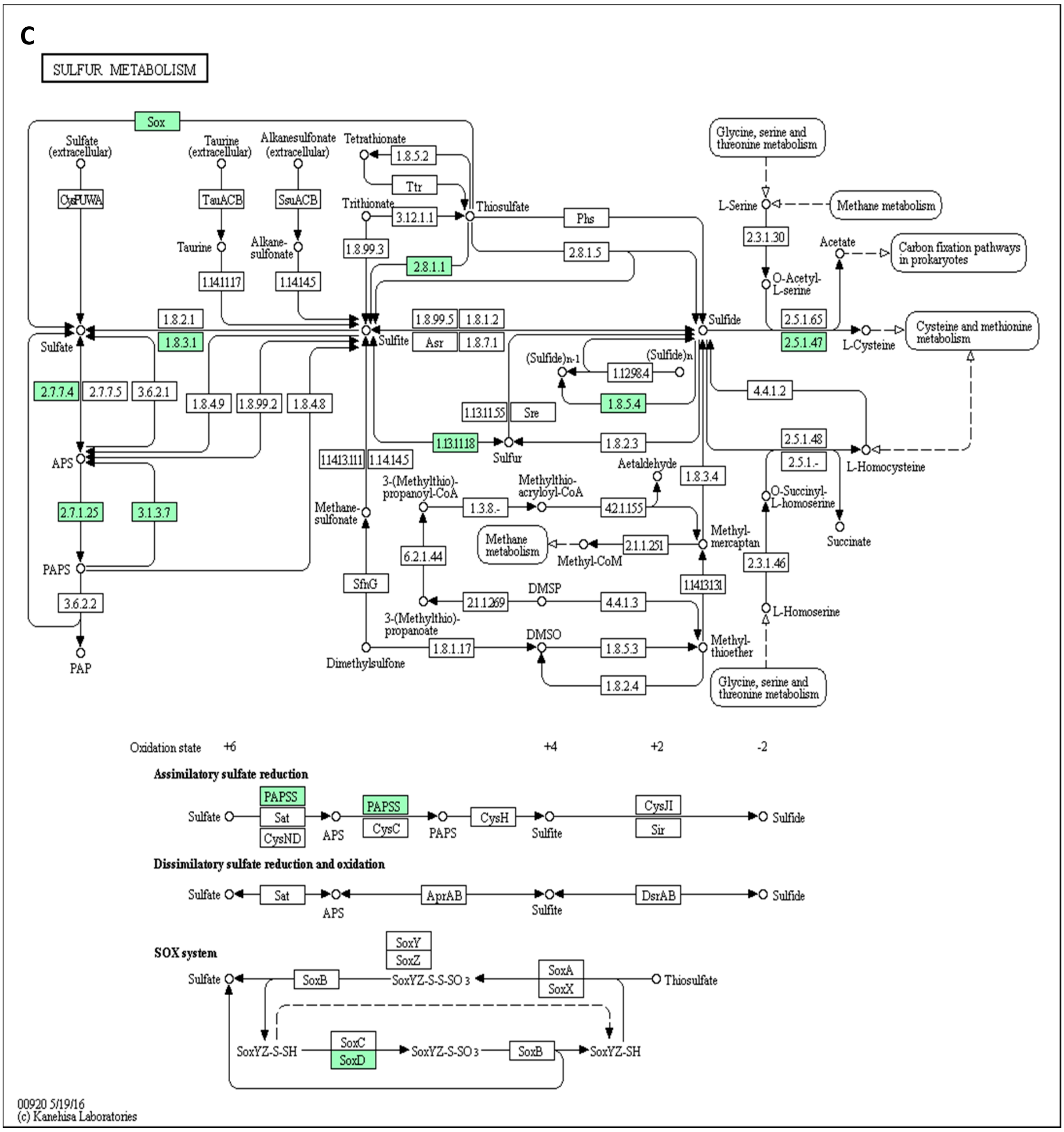

Figure S3. Sulfur metabolism pathways for (**A**) *Crassostrea gigas*, (**B**) *Lottia gigantea* and, (**C**) *Octopus bimaculoides*.

A

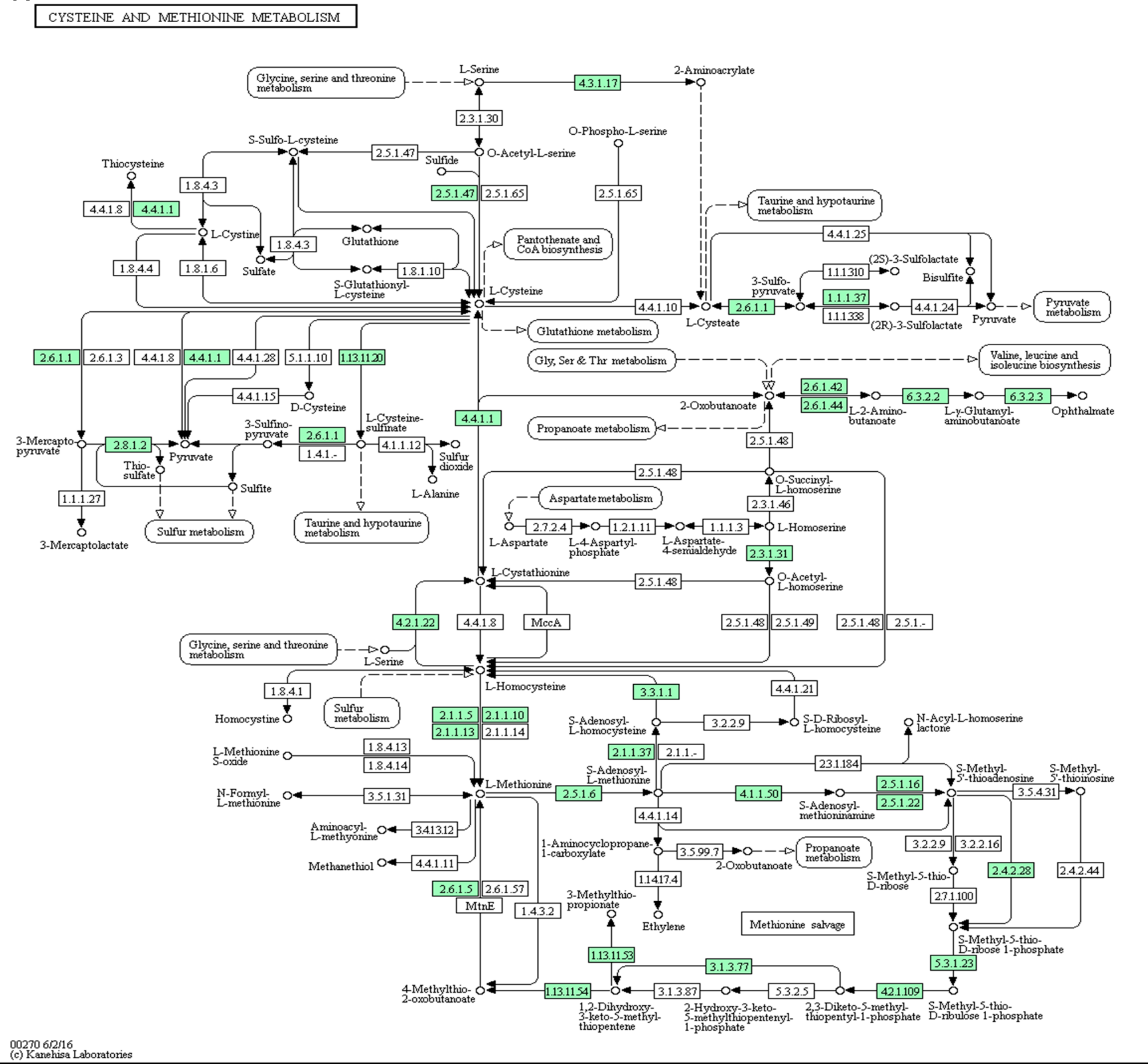
CYSTEINE AND METHIONINE METABOLISM
Glycine, serine and threonine metabolism
L-Serine
4.3.1.17
2-Aminoacrylate
2.3.1.30
O-Phospho-L-serine
S-Sulfo-L-cysteine
2.5.1.47
O-Acetyl-L-serine
Sulfide
Thiocysteine
1.8.4.3
4.4.1.8
4.4.1.1
2.5.1.47
2.5.1.65
2.5.1.65
Taurine and hypotaurine metabolism
L-Cystine
1.8.4.3
Glutathione
Pantothenate and CoA biosynthesis
4.4.1.25
1.8.4.4
1.8.1.6
Sulfate
1.8.1.10
S-Glutathionyl-L-cysteine
L-Cysteine
(2S)-3-Sulfolactate
1.1.1.310
Bisulfite
3-Sulfo-pyruvate
1.1.1.37
4.4.1.10
2.6.1.1
1.1.1.338
4.4.1.24
Pyruvate
Pyruvate metabolism
L-Cysteate
(2R)-3-Sulfolactate
Glutathione metabolism
2.6.1.1
2.6.1.3
4.4.1.8
4.4.1.1
4.4.1.28
5.1.1.10
1.13.11.20
Gly, Ser & Thr metabolism
Valine, leucine and isoleucine biosynthesis
2.6.1.42
2.6.1.44
6.3.2.2
6.3.2.3
4.4.1.15
D-Cysteine
2-Oxobutanoate
L-2-Amino-butanoate
L-γ-Glutamyl-aminobutanoate
Ophthalmate
4.4.1.1
3-Sulfino-pyruvate
L-Cysteine-sulfinate
Propanoate metabolism
2.6.1.1
3-Mercapto-pyruvate
2.8.1.2
Pyruvate
4.1.1.12
1.4.1.-
Sulfur dioxide
2.5.1.48
Thio-sulfate
Sulfite
L-Alanine
2.5.1.48
O-Succinyl-L-homoserine
1.1.1.27
Aspartate metabolism
2.3.1.46
Sulfur metabolism
Taurine and hypotaurine metabolism
2.7.2.4
1.2.1.11
1.1.1.3
L-Homoserine
3-Mercaptolactate
L-Aspartate
L-4-Aspartyl-phosphate
L-Aspartate-4-semialdehyde
2.3.1.31
L-Cystathionine
2.5.1.48
O-Acetyl-L-homoserine
4.2.1.22
4.4.1.8
MccA
2.5.1.48
2.5.1.49
2.5.1.48
2.5.1.-
Glycine, serine and threonine metabolism
L-Serine
L-Homocysteine
1.8.4.1
Sulfur metabolism
3.3.1.1
4.4.1.21
Homocystine
2.1.1.5
2.1.1.10
S-Adenosyl-L-homocysteine
3.2.2.9
S-D-Ribosyl-L-homocysteine
N-Acyl-L-homoserine lactone
2.1.1.13
2.1.1.14
L-Methionine S-oxide
1.8.4.13
1.8.4.14
2.1.1.37
2.1.1.-
2.3.1.184
S-Adenosyl-L-methionine
S-Methyl-5'-thioadenosine
S-Methyl-5'-thioinosine
N-Formyl-L-methionine
3.5.1.31
L-Methionine
2.5.1.6
4.1.1.50
2.5.1.16
3.5.4.31
2.5.1.22
S-Adenosyl-methioninamine
4.4.1.14
Aminoacyl-L-methyonine
3.4.13.12
3.2.2.9
3.2.2.16
1-Aminocyclopropane-1-carboxylate
3.5.99.7
Propanoate metabolism
Methanethiol
4.4.1.11
2-Oxobutanoate
2.4.2.28
2.4.2.44
S-Methyl-5-thio-D-ribose
1.14.17.4
2.6.1.5
2.6.1.57
3-Methylthio-propionate
2.7.1.100
MtnE
1.4.3.2
Ethylene
Methionine salvage
S-Methyl-5-thio-D-ribose 1-phosphate
1.13.11.53
5.3.1.23
3.1.3.77
4-Methylthio-2-oxobutanoate
1.13.11.54
3.1.3.87
5.3.2.5
4.2.1.109
1,2-Dihydroxy-3-keto-5-methyl-thiopentene
2-Hydroxy-3-keto-5-methylthiopentenyl-1-phosphate
2,3-Diketo-5-methyl-thiopentyl-1-phosphate
S-Methyl-5-thio-D-ribulose 1-phosphate
00270 6/2/16
(c) Kanehisa Laboratories

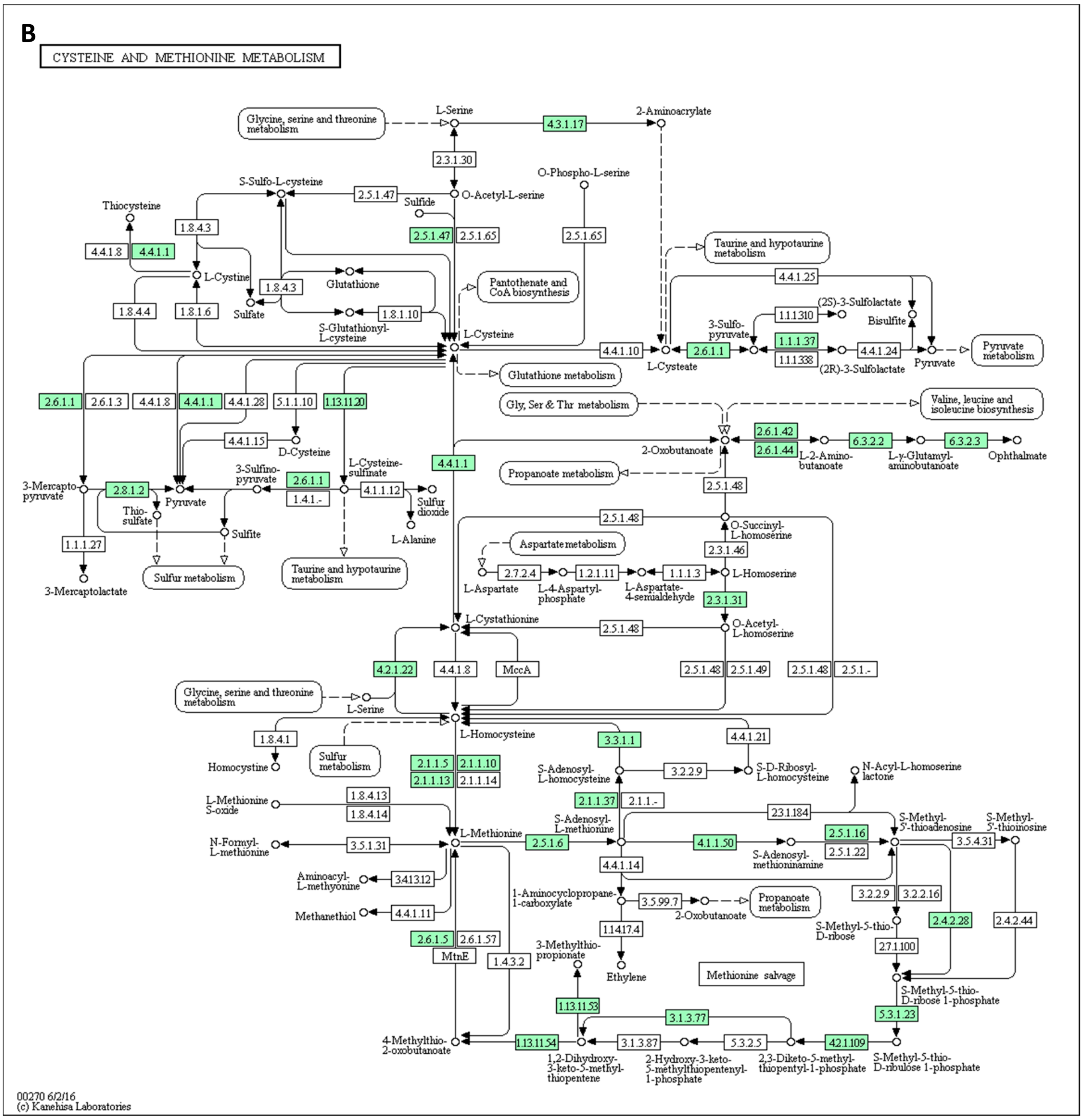
B
CYSTEINE AND METHIONINE METABOLISM
Glycine, serine and threonine metabolism
L-Serine
4.3.1.17
2-Aminoacrylate
2.3.1.30
O-Phospho-L-serine
S-Sulfo-L-cysteine
2.5.1.47
O-Acetyl-L-serine
Sulfide
Thiocysteine
1.8.4.3
4.4.1.8
4.4.1.1
2.5.1.47
2.5.1.65
2.5.1.65
Taurine and hypotaurine metabolism
L-Cystine
1.8.4.3
Glutathione
Pantothenate and CoA biosynthesis
4.4.1.25
1.8.4.4
1.8.1.6
Sulfate
1.8.1.10
S-Glutathionyl-L-cysteine
L-Cysteine
1.1.1.310
(2S)-3-Sulfolactate
Bisulfite
3-Sulfo-pyruvate
1.1.1.37
1.1.1.338
(2R)-3-Sulfolactate
4.4.1.10
L-Cysteate
2.6.1.1
4.4.1.24
Pyruvate
Pyruvate metabolism
Glutathione metabolism
2.6.1.1
2.6.1.3
4.4.1.8
4.4.1.1
4.4.1.28
5.1.1.10
1.13.11.20
Gly, Ser & Thr metabolism
Valine, leucine and isoleucine biosynthesis
4.4.1.15
D-Cysteine
2-Oxobutanoate
2.6.1.42
2.6.1.44
L-2-Amino-butanoate
6.3.2.2
L-γ-Glutamyl-aminobutanoate
6.3.2.3
Ophthalmate
4.4.1.1
Propanoate metabolism
3-Sulfino-pyruvate
L-Cysteine-sulfinate
3-Mercapto-pyruvate
2.8.1.2
Pyruvate
2.6.1.1
4.1.1.12
Sulfur dioxide
1.4.1.-
Thio-sulfate
Sulfite
L-Alanine
2.5.1.48
1.1.1.27
Sulfur metabolism
Taurine and hypotaurine metabolism
3-Mercaptolactate
O-Succinyl-L-homoserine
Aspartate metabolism
2.3.1.46
2.7.2.4
1.2.1.11
1.1.1.3
L-Aspartate
L-4-Aspartyl-phosphate
L-Aspartate-4-semialdehyde
L-Homoserine
2.3.1.31
L-Cystathionine
2.5.1.48
O-Acetyl-L-homoserine
4.2.1.22
4.4.1.8
MccA
2.5.1.48
2.5.1.49
2.5.1.48
2.5.1.-
Glycine, serine and threonine metabolism
L-Serine
L-Homocysteine
1.8.4.1
3.3.1.1
4.4.1.21
Homocystine
Sulfur metabolism
2.1.1.5
2.1.1.10
2.1.1.13
2.1.1.14
S-Adenosyl-L-homocysteine
3.2.2.9
S-D-Ribosyl-L-homocysteine
N-Acyl-L-homoserine lactone
L-Methionine S-oxide
1.8.4.13
1.8.4.14
2.1.1.37
2.1.1.-
2.3.1.184
S-Adenosyl-L-methionine
S-Methyl-5'-thioadenosine
S-Methyl-5'-thioinosine
N-Formyl-L-methionine
3.5.1.31
L-Methionine
2.5.1.6
4.1.1.50
S-Adenosyl-methioninamine
2.5.1.16
2.5.1.22
3.5.4.31
Aminoacyl-L-methionine
3.4.13.12
4.4.1.14
Methanethiol
4.4.1.11
1-Aminocyclopropane-1-carboxylate
3.5.99.7
2-Oxobutanoate
Propanoate metabolism
3.2.2.9
3.2.2.16
2.6.1.5
2.6.1.57
1.14.17.4
S-Methyl-5-thio-D-ribose
2.4.2.28
2.4.2.44
MtnE
1.4.3.2
3-Methylthio-propionate
2.7.1.100
Ethylene
Methionine salvage
S-Methyl-5-thio-D-ribose 1-phosphate
1.13.11.53
3.1.3.77
5.3.1.23
4-Methylthio-2-oxobutanoate
1.13.11.54
3.1.3.87
5.3.2.5
4.2.1.109
1,2-Dihydroxy-3-keto-5-methylthiopentene
2-Hydroxy-3-keto-5-methylthiopentenyl-1-phosphate
2,3-Diketo-5-methylthiopentyl-1-phosphate
S-Methyl-5-thio-D-ribulose 1-phosphate
00270 6/2/16
(c) Kanehisa Laboratories

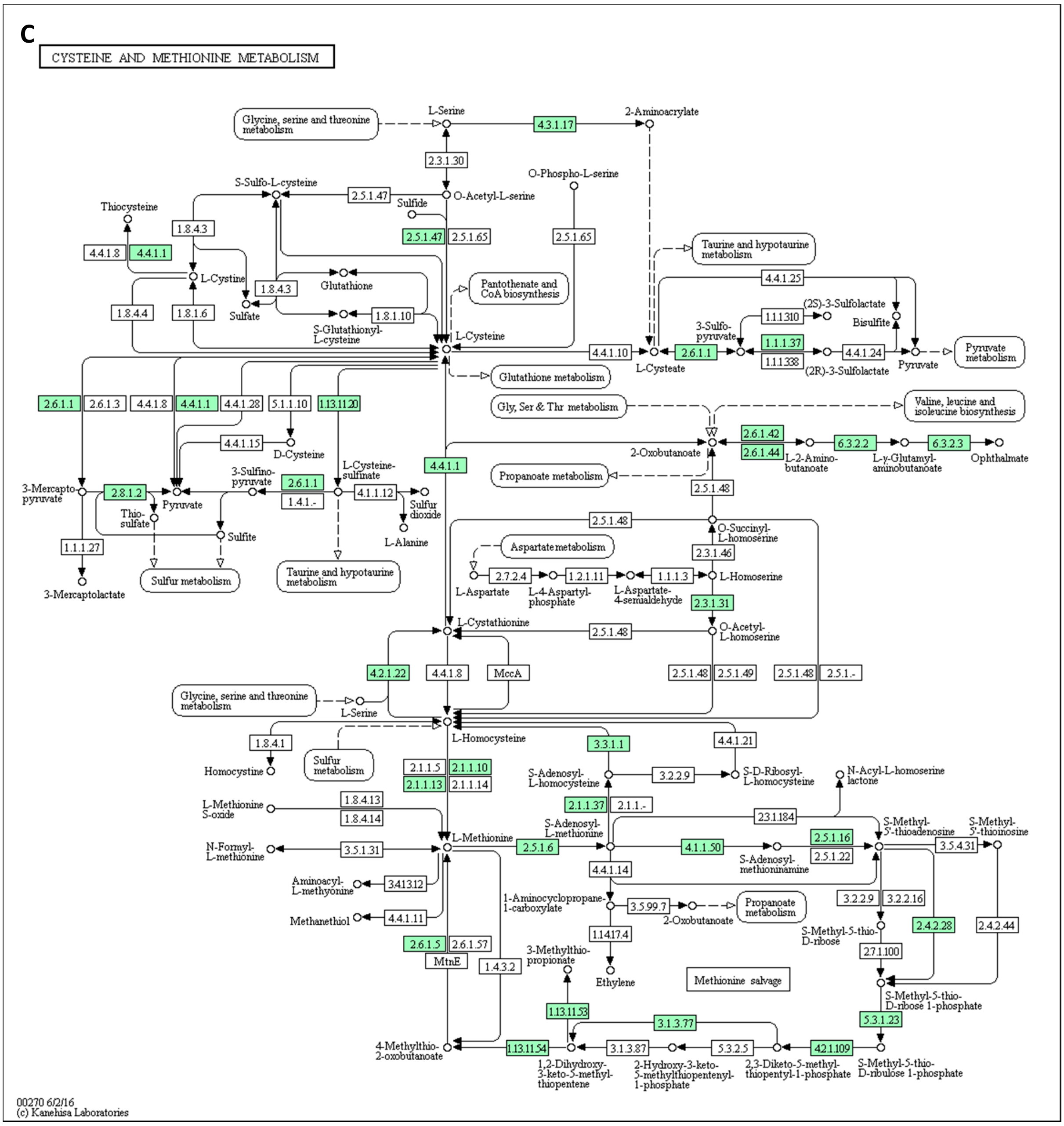

Figure S4. Cysteine and methionine metabolism pathway of (**A**) *Crassostrea gigas*, (**B**) *Lottia gigantea* and, (**C**) *Octopus bimaculoides*.

A

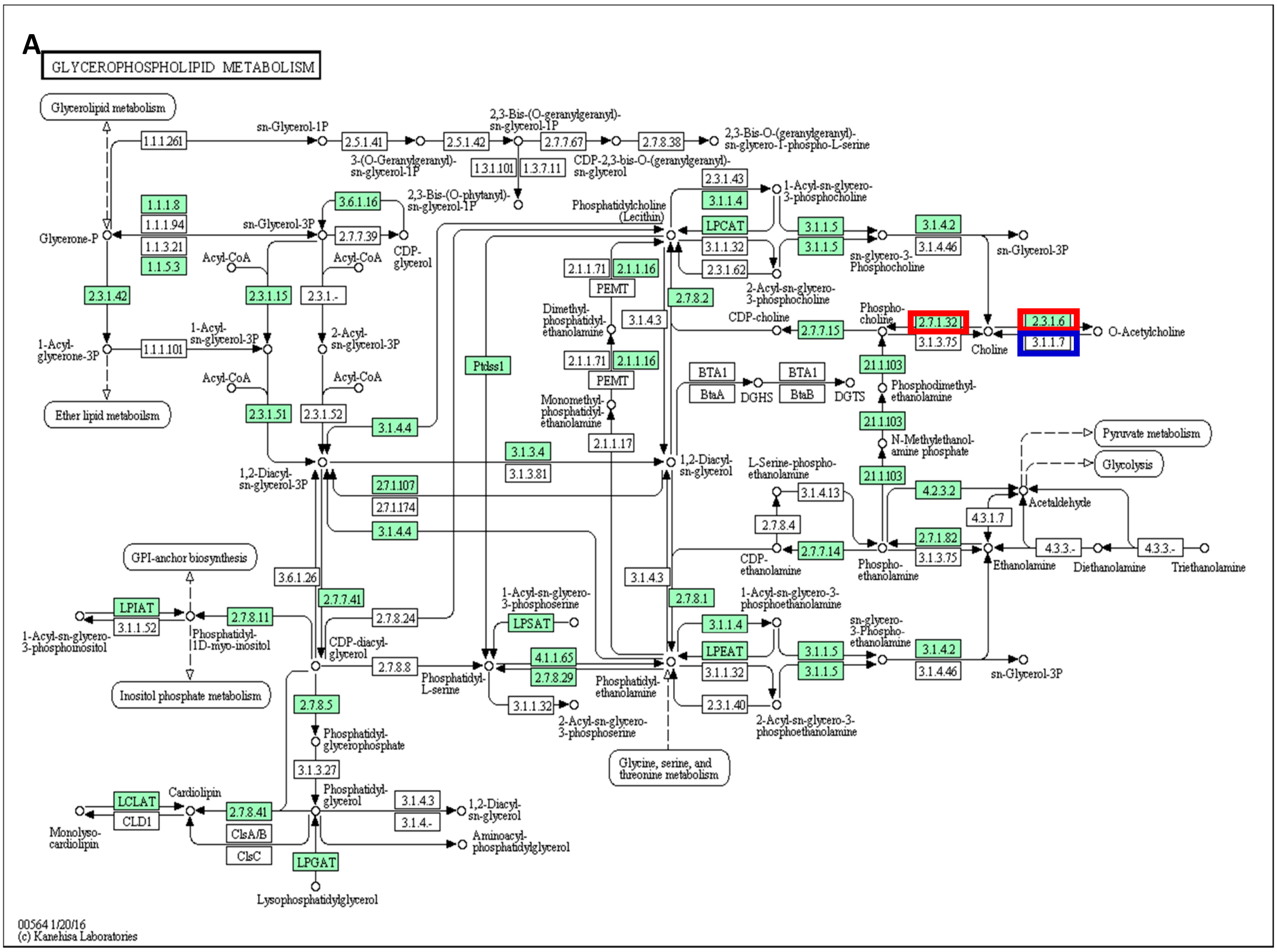
GLYCEROPHOSPHOLIPID METABOLISM
Glycerolipid metabolism
Ether lipid metabolism
GPI-anchor biosynthesis
Inositol phosphate metabolism
Glycine, serine, and threonine metabolism
Pyruvate metabolism
Glycolysis
sn-Glycerol-1P
2,3-Bis-(O-geranylgeranyl)-sn-glycerol-1P
3-(O-Geranylgeranyl)-sn-glycerol-1P
2,3-Bis-O-(geranylgeranyl)-sn-glycero-1-phospho-L-serine
CDP-2,3-bis-O-(geranylgeranyl)-sn-glycerol
2,3-Bis-(O-phytanyl)-sn-glycerol-1P
Glycerone-P
sn-Glycerol-3P
Acyl-CoA
CDP-glycerol
1-Acyl-glycerone-3P
1-Acyl-sn-glycerol-3P
2-Acyl-sn-glycerol-3P
1,2-Diacyl-sn-glycerol-3P
Phosphatidylcholine (Lecithin)
1-Acyl-sn-glycero-3-phosphocholine
2-Acyl-sn-glycero-3-phosphocholine
sn-glycero-3-Phosphocholine
sn-Glycerol-3P
CDP-choline
Phosphocholine
Choline
O-Acetylcholine
Dimethyl-phosphatidyl-ethanolamine
Monomethyl-phosphatidyl-ethanolamine
Phosphodimethyl-ethanolamine
N-Methylethanol-amine phosphate
DGHS
DGTS
1,2-Diacyl-sn-glycerol
L-Serine-phospho-ethanolamine
Acetaldehyde
CDP-ethanolamine
Phospho-ethanolamine
Ethanolamine
Diethanolamine
Triethanolamine
1-Acyl-sn-glycero-3-phosphoserine
1-Acyl-sn-glycero-3-phosphoethanolamine
sn-glycero-3-Phospho-ethanolamine
CDP-diacyl-glycerol
Phosphatidyl-L-serine
Phosphatidyl-ethanolamine
2-Acyl-sn-glycero-3-phosphoserine
2-Acyl-sn-glycero-3-phosphoethanolamine
1-Acyl-sn-glycero-3-phosphoinositol
Phosphatidyl-1D-myo-inositol
Phosphatidyl-glycerophosphate
Phosphatidyl-glycerol
Cardiolipin
Monolyso-cardiolipin
Aminoacyl-phosphatidylglycerol
Lysophosphatidylglycerol
1.1.1.261
2.5.1.41
2.5.1.42
2.7.7.67
2.7.8.38
1.3.1.101
1.3.7.11
1.1.1.8
1.1.1.94
1.1.3.21
1.1.5.3
3.6.1.16
2.7.7.39
2.3.1.42
2.3.1.15
2.3.1.-
1.1.1.101
2.3.1.51
2.3.1.52
3.1.4.4
2.3.1.43
3.1.1.4
LPCAT
3.1.1.32
2.3.1.62
3.1.1.5
3.1.4.2
3.1.4.46
2.1.1.71
2.1.1.16
PEMT
2.7.8.2
3.1.4.3
2.7.7.15
2.7.1.32
3.1.3.75
2.3.1.6
3.1.1.7
Ptdss1
BTA1
BtaA
BtaB
2.1.1.103
2.1.1.17
3.1.3.4
3.1.3.81
2.7.1.107
2.7.1.174
3.1.4.13
2.7.8.4
4.2.3.2
4.3.1.7
2.7.7.14
2.7.1.82
4.3.3.-
3.6.1.26
2.7.7.41
2.7.8.24
LPIAT
3.1.1.52
2.7.8.11
LPSAT
2.7.8.1
LPEAT
4.1.1.65
2.7.8.29
2.7.8.8
3.1.1.32
2.3.1.40
2.7.8.5
3.1.3.27
LCLAT
CLD1
2.7.8.41
ClsA/B
ClsC
LPGAT
3.1.4.-
00564 1/20/16
(c) Kanehisa Laboratories

B

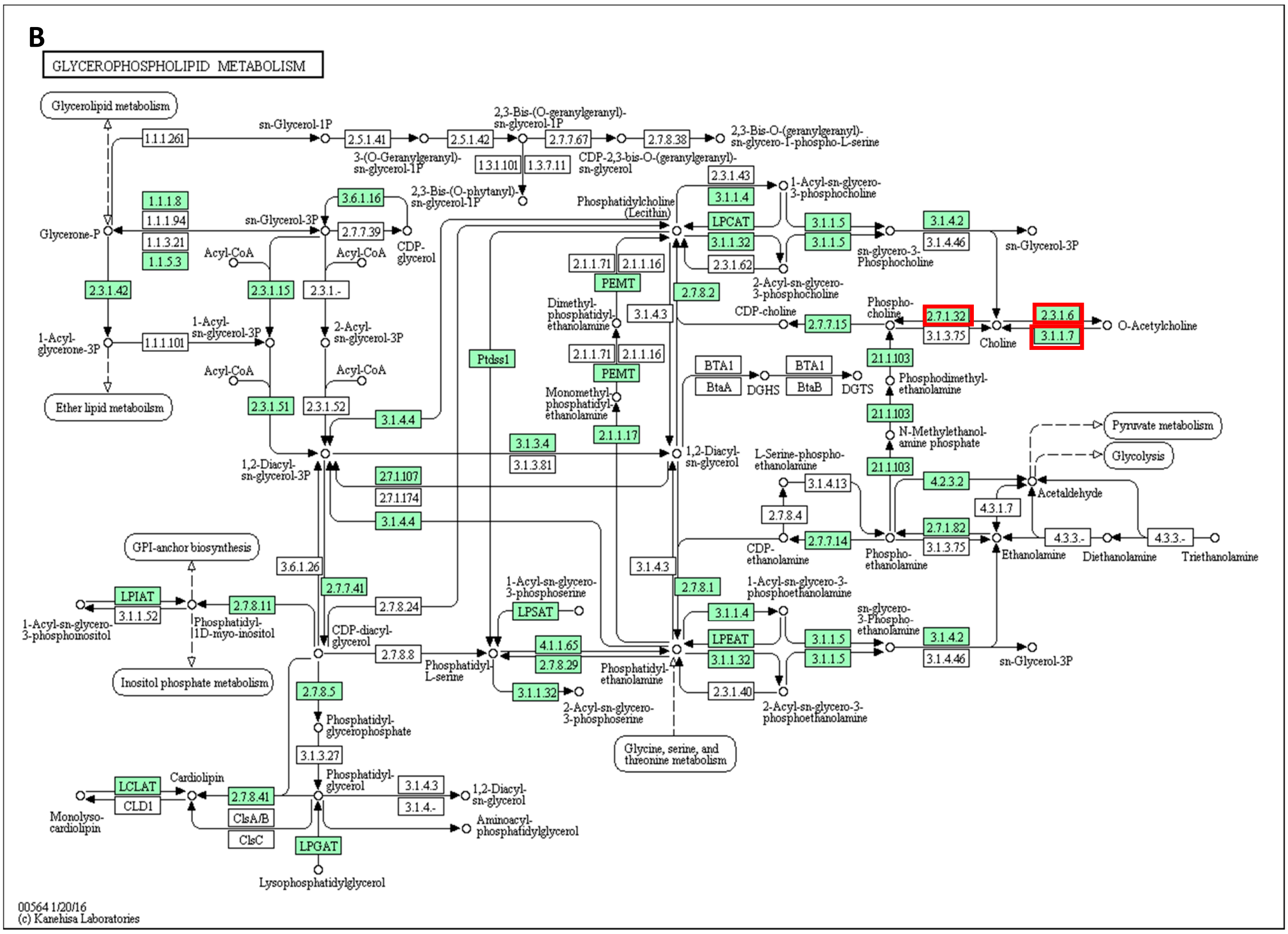
GLYCEROPHOSPHOLIPID METABOLISM
Glycerolipid metabolism
Ether lipid metabolism
GPI-anchor biosynthesis
Inositol phosphate metabolism
Glycine, serine, and threonine metabolism
Pyruvate metabolism
Glycolysis
sn-Glycerol-1P
2,3-Bis-(O-geranylgeranyl)-sn-glycerol-1P
3-(O-Geranylgeranyl)-sn-glycerol-1P
2,3-Bis-O-(geranylgeranyl)-sn-glycero-1-phospho-L-serine
CDP-2,3-bis-O-(geranylgeranyl)-sn-glycerol
2,3-Bis-(O-phytanyl)-sn-glycerol-1P
Glycerone-P
sn-Glycerol-3P
Acyl-CoA
CDP-glycerol
1-Acyl-glycerone-3P
1-Acyl-sn-glycerol-3P
2-Acyl-sn-glycerol-3P
1,2-Diacyl-sn-glycerol-3P
Phosphatidylcholine (Lecithin)
1-Acyl-sn-glycero-3-phosphocholine
2-Acyl-sn-glycero-3-phosphocholine
sn-glycero-3-Phosphocholine
CDP-choline
Phosphocholine
Choline
O-Acetylcholine
Dimethyl-phosphatidyl-ethanolamine
Monomethyl-phosphatidyl-ethanolamine
Phosphodimethyl-ethanolamine
N-Methylethanol-amine phosphate
DGHS
DGTS
1,2-Diacyl-sn-glycerol
L-Serine-phospho-ethanolamine
Acetaldehyde
CDP-ethanolamine
Phospho-ethanolamine
Ethanolamine
Diethanolamine
Triethanolamine
1-Acyl-sn-glycero-3-phosphoserine
1-Acyl-sn-glycero-3-phosphoethanolamine
sn-glycero-3-Phospho-ethanolamine
CDP-diacyl-glycerol
Phosphatidyl-L-serine
Phosphatidyl-ethanolamine
2-Acyl-sn-glycero-3-phosphoserine
2-Acyl-sn-glycero-3-phosphoethanolamine
1-Acyl-sn-glycero-3-phosphoinositol
Phosphatidyl-1D-myo-inositol
Phosphatidyl-glycerophosphate
Phosphatidyl-glycerol
Cardiolipin
Monolyso-cardiolipin
Aminoacyl-phosphatidylglycerol
Lysophosphatidylglycerol
00564 1/20/16
(c) Kanehisa Laboratories

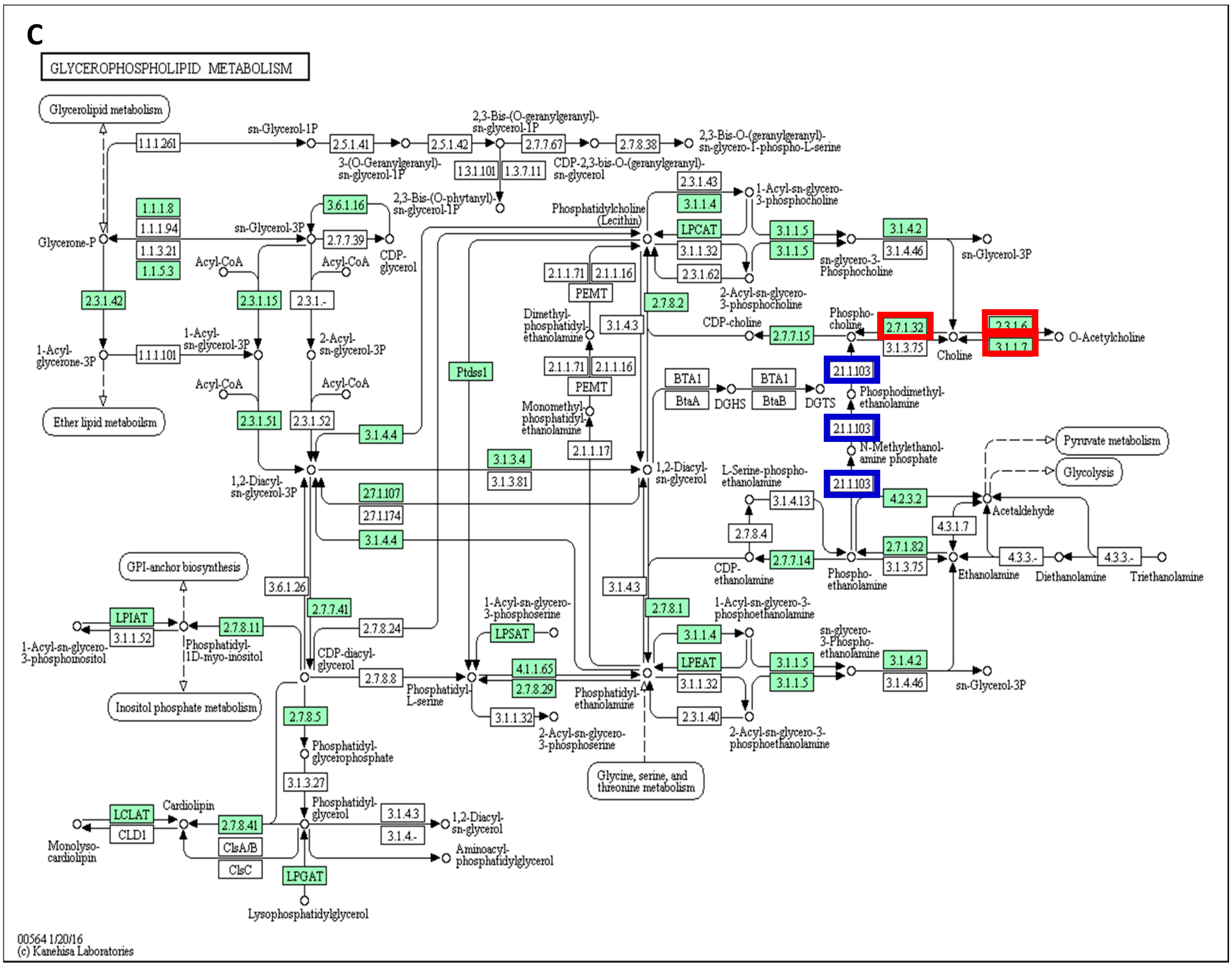

Figure S5. Glycerophospholipid metabolism pathway of (**A**) *Crassostrea gigas*, (**B**) *Lottia gigantea* and, (**C**) *Octopus bimaculoides* showing enzyme matches in green with those relevant to choline ester synthesis highlighted in red (present) and blue (absent).

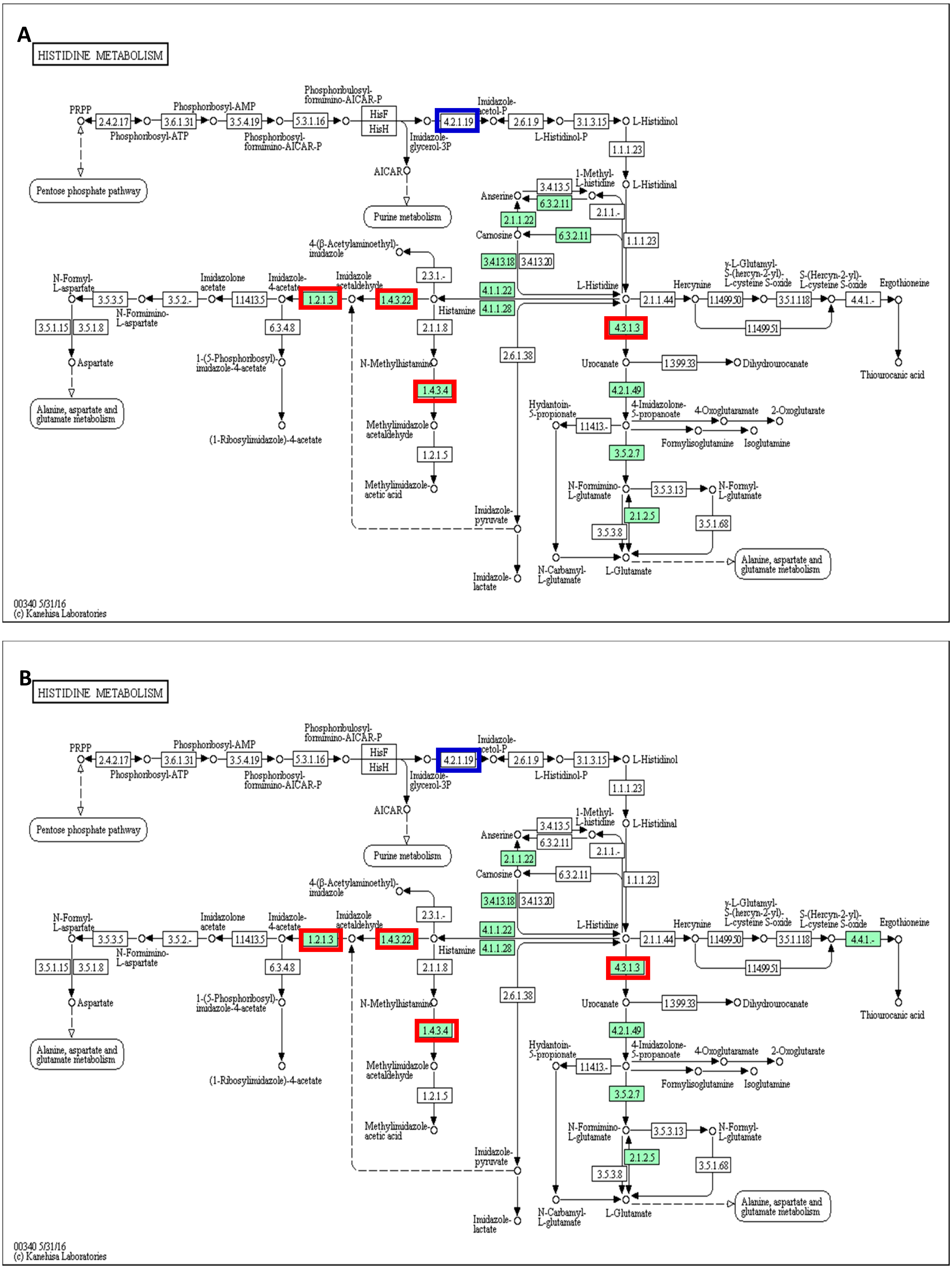
A
HISTIDINE METABOLISM
PRPP
2.4.2.17
Phosphoribosyl-AMP
3.6.1.31
Phosphoribosyl-ATP
3.5.4.19
Phosphoribosyl-formimino-AICAR-P
5.3.1.16
Phosphoribulosyl-formimino-AICAR-P
HisF
HisH
4.2.1.19
Imidazole-glycerol-3P
Imidazole-acetol-P
2.6.1.9
L-Histidinol-P
3.1.3.15
L-Histidinol
1.1.1.23
L-Histidinal
AICAR
Purine metabolism
Pentose phosphate pathway
1-Methyl-L-histidine
3.4.13.5
6.3.2.11
Anserine
2.1.1.-
2.1.1.22
Carnosine
6.3.2.11
1.1.1.23
3.4.13.18
3.4.13.20
4-(β-Acetylaminoethyl)-imidazole
2.3.1.-
4.1.1.22
4.1.1.28
L-Histidine
Histamine
N-Formyl-L-aspartate
3.5.3.5
N-Formimino-L-aspartate
3.5.2.-
Imidazolone acetate
1.14.13.5
Imidazole-4-acetate
1.2.1.3
Imidazole acetaldehyde
1.4.3.22
6.3.4.8
2.1.1.8
3.5.1.15
3.5.1.8
Aspartate
Alanine, aspartate and glutamate metabolism
1-(5-Phosphoribosyl)-imidazole-4-acetate
(1-Ribosylimidazole)-4-acetate
N-Methylhistamine
1.4.3.4
Methylimidazole acetaldehyde
1.2.1.5
Methylimidazole-acetic acid
2.6.1.38
Imidazole-pyruvate
Imidazole-lactate
Hercynine
2.1.1.44
1.14.99.50
γ-L-Glutamyl-S-(hercyn-2-yl)-L-cysteine S-oxide
3.5.1.118
S-(Hercyn-2-yl)-L-cysteine S-oxide
4.4.1.-
Ergothioneine
1.14.99.51
Thiourocanic acid
4.3.1.3
Urocanate
1.3.99.33
Dihydrourocanate
4.2.1.49
Hydantoin-5-propionate
1.14.13.-
4-Imidazolone-5-propanoate
4-Oxoglutaramate
2-Oxoglutarate
Formylisoglutamine
Isoglutamine
3.5.2.7
N-Formimino-L-glutamate
3.5.3.13
N-Formyl-L-glutamate
2.1.2.5
3.5.3.8
3.5.1.68
N-Carbamyl-L-glutamate
L-Glutamate
Alanine, aspartate and glutamate metabolism
00340 5/31/16
(c) Kanehisa Laboratories
B
HISTIDINE METABOLISM
PRPP
2.4.2.17
Phosphoribosyl-AMP
3.6.1.31
Phosphoribosyl-ATP
3.5.4.19
Phosphoribosyl-formimino-AICAR-P
5.3.1.16
Phosphoribulosyl-formimino-AICAR-P
HisF
HisH
4.2.1.19
Imidazole-glycerol-3P
Imidazole-acetol-P
2.6.1.9
L-Histidinol-P
3.1.3.15
L-Histidinol
1.1.1.23
L-Histidinal
AICAR
Purine metabolism
Pentose phosphate pathway
1-Methyl-L-histidine
3.4.13.5
6.3.2.11
Anserine
2.1.1.-
2.1.1.22
Carnosine
6.3.2.11
1.1.1.23
3.4.13.18
3.4.13.20
4-(β-Acetylaminoethyl)-imidazole
2.3.1.-
4.1.1.22
4.1.1.28
L-Histidine
Histamine
N-Formyl-L-aspartate
3.5.3.5
N-Formimino-L-aspartate
3.5.2.-
Imidazolone acetate
1.14.13.5
Imidazole-4-acetate
1.2.1.3
Imidazole acetaldehyde
1.4.3.22
6.3.4.8
2.1.1.8
3.5.1.15
3.5.1.8
Aspartate
Alanine, aspartate and glutamate metabolism
1-(5-Phosphoribosyl)-imidazole-4-acetate
(1-Ribosylimidazole)-4-acetate
N-Methylhistamine
1.4.3.4
Methylimidazole acetaldehyde
1.2.1.5
Methylimidazole-acetic acid
2.6.1.38
Imidazole-pyruvate
Imidazole-lactate
Hercynine
2.1.1.44
1.14.99.50
γ-L-Glutamyl-S-(hercyn-2-yl)-L-cysteine S-oxide
3.5.1.118
S-(Hercyn-2-yl)-L-cysteine S-oxide
4.4.1.-
Ergothioneine
1.14.99.51
Thiourocanic acid
4.3.1.3
Urocanate
1.3.99.33
Dihydrourocanate
4.2.1.49
Hydantoin-5-propionate
1.14.13.-
4-Imidazolone-5-propanoate
4-Oxoglutaramate
2-Oxoglutarate
Formylisoglutamine
Isoglutamine
3.5.2.7
N-Formimino-L-glutamate
3.5.3.13
N-Formyl-L-glutamate
2.1.2.5
3.5.3.8
3.5.1.68
N-Carbamyl-L-glutamate
L-Glutamate
Alanine, aspartate and glutamate metabolism
00340 5/31/16
(c) Kanehisa Laboratories

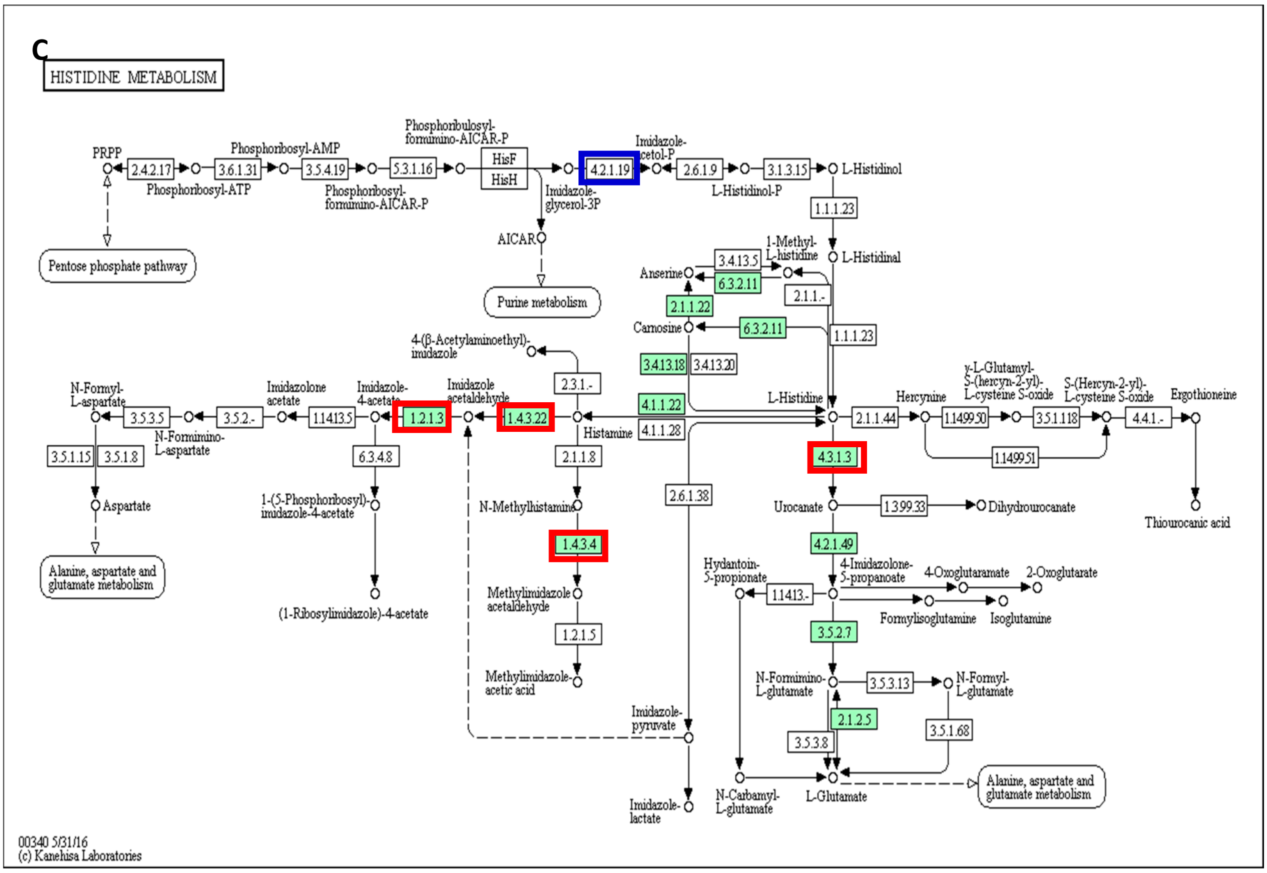

Figure S6. Histidine metabolism pathway of (**A**) *Crassostrea gigas*, (**B**) *Lottia gigantea* and, (**C**) *Octopus bimaculoides* showing matching enzymes in green, including several enzymes that convert histidine into imidazole (red boxes) but not imidazoleglycerol-phosphate dehydratase (blue box).
